# The Chemical Nature of the Oxide Directs the Stability and Reactivity of Copper|Oxide Interfaces in the Electrochemical CO_2_ Reduction Reaction

**DOI:** 10.1021/acs.chemmater.5c00135

**Published:** 2025-04-24

**Authors:** Jari Leemans, Jennifer Calderon Mora, Petru P. Albertini, Krishna Kumar, Coline Boulanger, Raffaella Buonsanti

**Affiliations:** Laboratory of Nanochemistry for Energy, Institute of Chemical Sciences and Engineering, École Polytechnique Fédérale de Lausanne, 1950 Sion, Switzerland

## Abstract

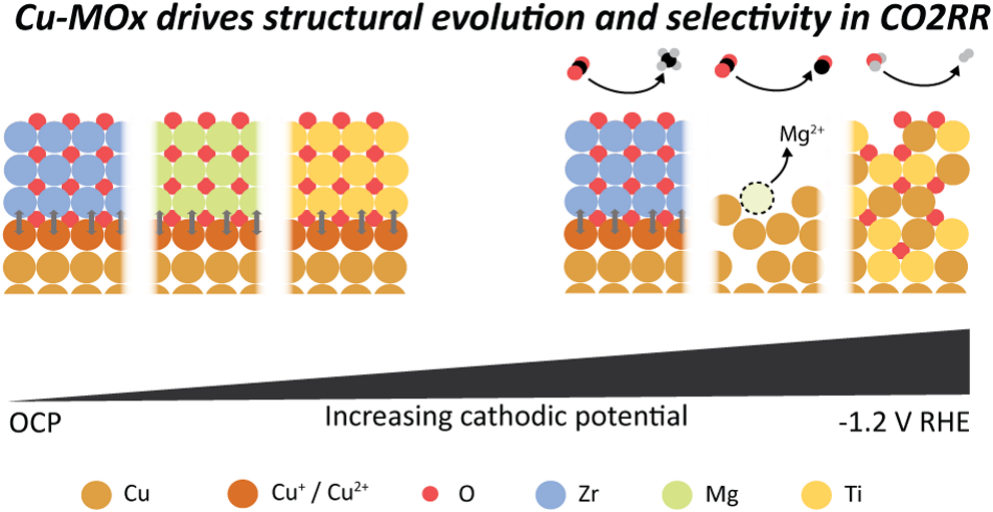

Understanding the impact of composition and interfaces between metals and oxides is a goal of interest for many chemical reactions. Herein, we propose a framework to map correlations between the electrochemical behavior of the oxide and the stability and reactivity of metal|oxide interfaces, exemplified by Cu|oxide for the electrochemical CO_2_ reduction reaction (CO_2_RR). Copper materials interfaced with metal oxides have emerged as promising CO_2_RR catalysts for selectivity toward multicarbon products, including alcohols; stability under operation has been reported for some of them. However, design rules are currently lacking. Herein, we propose the synthesis of well-defined Cu-MOx core–shell nanoparticles to investigate and compare the behavior of Cu-ZrOx, Cu-MgOx, and Cu-TiOx. By tracking the speciation and morphological evolution of these model catalyst materials, we find that the cathodic stability of the formed interfaces is determined by the operating potential and phase stability of the pure oxides and by their chemical interaction with copper. We learn that the interplay between these factors shapes the restructuring pathways for Cu-MOx catalysts and eventually drives their selectivity in the CO_2_RR. The developed understanding can be applied beyond this reaction, and the developed nanomaterials can be used beyond catalysis.

## Introduction

Developing design rules for active, selective, and stable catalysts is one of the major goals in catalysis science.^1−8^ Elucidating catalyst design rules demands for both the investigation of catalysts which do not possess the desirable reactivity and catalysts which are highly active and selective for a given reaction.^1−8^ However, comparison among different catalysts is meaningful only when property-determining features (i.e., composition, particle size, interfaces) are varied one at a time.^9^ Tailored nanomaterials offer the opportunity to correlate the role of one specific feature to the properties and, eventually, to optimize the final performance in the application of interest.^1−8,10−12^ Thus, catalysis science can tremendously benefit from the advancement of nanochemistry.

The electrochemical CO_2_ reduction reaction (CO_2_RR) is currently one of the most investigated reactions.^7,13−18^ Studies on more selective and, to some extent, more stable catalysts have significantly increased over the past decade and continue to pile up in the literature.^7,17,19−27^ Yet, design rules remain unclear.

Copper materials interfaced with metal oxides have recently emerged as a promising class of CO_2_RR catalysts in terms of selectivity toward desirable alcohols and hydrocarbons and in terms of stability of the generated active sites.^17,20,28−31^ Record faradaic efficiencies for alcohols (Cu-BaOx),^20^ high multicarbon product (C_2+_) selectivity (Cu-ZrOx),^32^ and unprecedented morphological stability of Cu electrodes (Cu-AlOx)^17^ all make use of interfaced metal oxides to tune the catalytic behavior of copper.

However, different behaviors for the same Cu|MOx interface have been reported across the literature. For example, Cu-CeOx catalysts have been reported to be selective for methane or ethylene depending on the utilized synthesis methods and resulting composition and/or structure of the catalysts.^33−36^ A similar discrepancy arises with either ethanol from CO_2_RR or HER promotion over Cu-TiOx catalysts,^37−40^ or ethylene and ethanol on Cu–Mg bimetallic catalysts.^28,41^ On the contrary, reports on Cu-ZrOx demonstrate high C_2+_ products with a variety of catalyst preparation methods, and thus catalyst structures.^32,33,42^ Despite performant catalysis, these examples emphasize a lack of design rules that identify the catalysis-determining structural features. Strikingly, no discussion is provided on the evolution of the complex catalysts during operation, which, alongside varied test conditions, obscures a fundamental understanding of the correlations among the chemical nature of the oxide, the stability of the involved interfaces, and, finally, the reactivity.

Herein, we propose the chemistry to synthesize Cu-MOx core–shell nanostructures where size-controlled Cu domains are interfaced with various oxides (Ti, Mg, Zr) obtained by the same colloidal atomic layer deposition strategy. Through comparison of these tailored nanomaterials with physical mixtures, we reveal the importance of atomic interfaces in determining the different reactivity for CO_2_RR. Then, we study the electronic effects induced on the Cu by the shell oxides, as these effects are commonly used to explain the CO_2_RR performance of copper materials interfaced with metal oxides. We determine that electronic effects alone are insufficient to clarify the catalytic behavior, so we investigate the fate of the core–shell structures during operation. By doing so, we elucidate that the cathodic stability of the formed interfaces depends on an intricate balance between the thermodynamic stability of the pure oxide phases and the interfacial Cu–O–M bond strength. Altogether, these results provide a complete picture regarding the relationship between the nature of the oxide and the reactivity in the CO_2_RR observed for the different Cu-MOx materials, which we trace back to the phase stability of the passivating oxides and their interaction with the Cu domains.

## Experimental Section

### Chemicals

Copper(I) acetate (Cu(I)OAc, 98%), CuBr, tri-n-octylamine (technical grade, 98%), tri-*n*-octylphosphine oxide, oleic acid (technical grade, 90%), tetradecylphosphonic acid (97%), tetrakisdimethylamidotitanium, toluene (anhydrous, 99.8%), hydrogen peroxide (50%), 1,4-dioxane (anhydrous), and ethanol (absolute, anhydrous) were purchased from Sigma-Aldrich Chemie GmbH. Cyclopentadienyl magnesium (99.99% Mg) and tetrakisdimethylamidozirconium (98%, 99.99% Zr) were purchased from ABCR Swiss AG.

### Colloidal Atomic Layer Deposition of Metal Oxides on Cu Nanospheres

Cu nanospheres were synthesized and oxidized according to previous methods described in the Supporting Information. 20 μmol of oxidized Cu nanocrystals as determined by inductively coupled plasma optical emission spectrometry (ICP-OES) is diluted with 9 mL of anhydrous toluene in a scintillation vial. The scintillation vial is connected to the Schlenk line with the use of a needle and kept under a slight overpressure. Meanwhile, dilutions of the desired metal precursors (MR_n_ = tetrakis(dimethylamido)zirconium, tetrakis(dimethylamidotitanium, cyclopentadienyl magnesium)) to yield solutions of 0.32 mM (solution A) and 1.6 mM (solution B) in toluene are prepared in the glovebox. Stoichiometric solutions of H_2_O in dioxane are prepared by consecutive dilutions of water in dry dioxane to yield a solution of 0.64 (solution A) and 3.2 mM (solution B).

First, the dilute A solutions of metal precursor and water are charged into separate, dried, N_2_-flushed gastight syringes. With the use of a syringe pump, the metal precursor is injected at a rate of 0.02 mL/min. After injecting 0.2 mL, the wet dioxane solution is co-titrated to grow the respective oxide. 5 mL of the A solutions is titrated to the Cu nanocrystals. Afterward, the syringes are charged with the B solutions and 4.5 mL of each solution is titrated in total to the Cu nanocrystals. The reaction is terminated by injecting 100 μL of a 4 mM oleic acid solution to coordinate unreacted metal precursor. The reaction product is isolated by adding EtOH to the toluene solution and centrifuging. The product pellet is redispersed in toluene.

### c-ALD on Cu Nanocubes

For growth of metal oxide shells on Cu nanocubes, a similar procedure was used as for the spheres, but only the A solutions above are used. Similarly, an oxidative treatment with hydrogen peroxide was performed with a H_2_O_2_ stoichiometry corresponding to the moles of surface atoms in the Cu cubes sample. Opposed to the targeted 30% mole fraction of the titrated metal precursor in the case of 7 nm spheres, a mole fraction of 2% was targeted to attain a similar surface coverage on the larger cubes. 55 μmol of Cu in the form of Cu cubes were dispersed in 12 mL of dry toluene. After titrating the first 0.2 mL of the metal precursor A solution, 4.5 mL of both A solutions was co-titrated at a rate of 0.2 mL/min. The reaction was terminated by injecting 100 μL of a 4 mM oleic acid solution to coordinate unreacted metal precursor. The cubes were isolated via centrifugation and redispersion in toluene without the addition of EtOH.

### Electrochemical Experiments

Electrochemistry was performed in a custom polycarbonate H-type cell with an internal volume of 4 mL, where the catholyte and anolyte are separated by a Selemion AMV anion-exchange membrane. The cathode and anode are oriented in a parallel fashion. 0.1 M KHCO_3_ solution was used as the electrolyte. A Biologic SP-300 potentiostat was connected to the glassy carbon working electrode, the platinum foil counter electrode, and the Ag/AgCl reference electrode. CO_2_ was bubbled through both anolyte and catholyte compartments with a frit-fitted gas inlet. To study the synthesized nanomaterials, thin films were prepared by drop-casting 15 μg of Cu onto the 1.33 cm^2^ active area of the glassy carbon working electrode.

Samples were tested for the CO_2_ reduction reaction with the following protocol. First, electrochemical impedance spectroscopy was performed to estimate the uncompensated resistance in the cell. Dynamic *iR*-compensation at 85% was consequently applied to all experiments. Then, the sample was preconditioned with a single linear sweep voltammetry scan from open-circuit potential to −2 V vs Ag/AgCl. After a waiting period of 2 min, a series of cyclic voltammetry experiments were performed with a scan window of 100 mV around −0.4 V vs Ag/AgCl and increasing scan rates to calculate the double-layer capacitance, from which the electrochemical surface area can be estimated. Then, 1 h of chronoamperometry at the desired potential was performed with a constant CO_2_ flow rate of 5 sccm. Following the chronoamperometry, another double-layer experiment was performed to estimate the double-layer capacitance after electrolysis.

Gas products were separated and analyzed with the use of a gas chromatograph (GC, SRI Instruments) equipped with a HayeSep D porous polymer column, thermal conductivity, and flame ionization detectors. The exhaust of the catholyte compartment is directly connected to the inlet of the GC. Liquid products are isolated by sampling the electrolyte postelectrolysis. Liquid products are separated and quantified with the use of high-performance liquid chromatography and a refractive index detector.

### Electron Microscopy

Sample preparation of the purified nanoparticles consisted of drop-casting 10 μL of the respective dispersions onto a Cu grid. Postelectrolysis electron microscopy sample preparation was performed by rinsing the electrode with water, consequently drop-casting 10 μL of toluene onto the dry electrode surface and scraping the catalyst residue onto the TEM grid through the toluene. Bright-field electron microscopy was performed on a Thermo Fisher Scientific Tecnai G2 Spirit Twin at 120 kV equipped with a Lens-coupled 4 megapixel Gatan Orius SC200D camera. High-angle annular dark-field scanning transmission electron microscopy and energy-dispersive X-ray spectroscopy were performed on a Thermo Fisher Scientific Spectra200 microscope at 200 kV. The elemental maps are created from the integrated intensity of the Cu K, Ti K, Zr L and Zr K, and Mg K line emission following background correction with the Velox software.

### X-ray Photoelectron Spectroscopy

XPS spectra were recorded using an Axis Supra (Kratos Analytical) instrument using the monochromated Kα X-ray line of an Al anode. The pass energy was set to 20 eV with a step size of 0.1 eV. The samples were electrically insulated from the sample holder, and charges were compensated. Spectra were calibrated to 284.8 eV at the C 1s orbital. Cu NCs samples were prepared by drop-casting nanocrystal films onto clean Si substrates.

## Results

### Materials Chemistry Development via Colloidal Atomic Layer Deposition (c-ALD)

We targeted a series of Cu-MOx with well-defined and similar composition and size to facilitate comparison to each other and physical mixtures of equal composition with the goal of studying the behavior of interfaces between Cu and metal oxides. We chose to explore zirconium, titanium, and magnesium oxide. These oxides are chemically very distinct, including their electrochemical phase stability according to the Pourbaix diagrams and the possibility to interface or even alloy with Cu, which are both relevant properties for their fate under CO_2_RR operating conditions.^43−45^ These oxides interfaced with Cu have been previously explored for CO_2_RR and contrasting results have been reported with regard to product selectivity and stability of the generated active sites.^28,32,33,39,40,42,46,47^ Currently, no synthesis pathways exist to synthesize Cu-MOx nanostructures with well-defined size below 10 nm, narrow size distribution, and uniform morphology across different compositions.^28,32,33,39,40,42,46,47^

Following previous results demonstrating the impressive structural stability of Cu nanocrystals coated with amorphous aluminum oxide,^17^ we decided to expand the same chemistry to other oxides. This chemistry is based on the colloidal atomic layer deposition (c-ALD) method.^17,48−51^ The chemistry of c-ALD lies in between that of gas-phase ALD and that of molecular layer deposition because of the inorganic/organic nature of the colloidal nanocrystal surface.^17,48−53^ We expect the same precursors used in gas-phase ALD to be suitable for c-ALD. Yet, the successful coating of the copper nanospheres with oxides requires a delicate balance between the oxide nucleation and growth and the colloidal stability, which needs to be preserved via organic ligands binding to the surface.^17,48−51^

**Figure 1:**
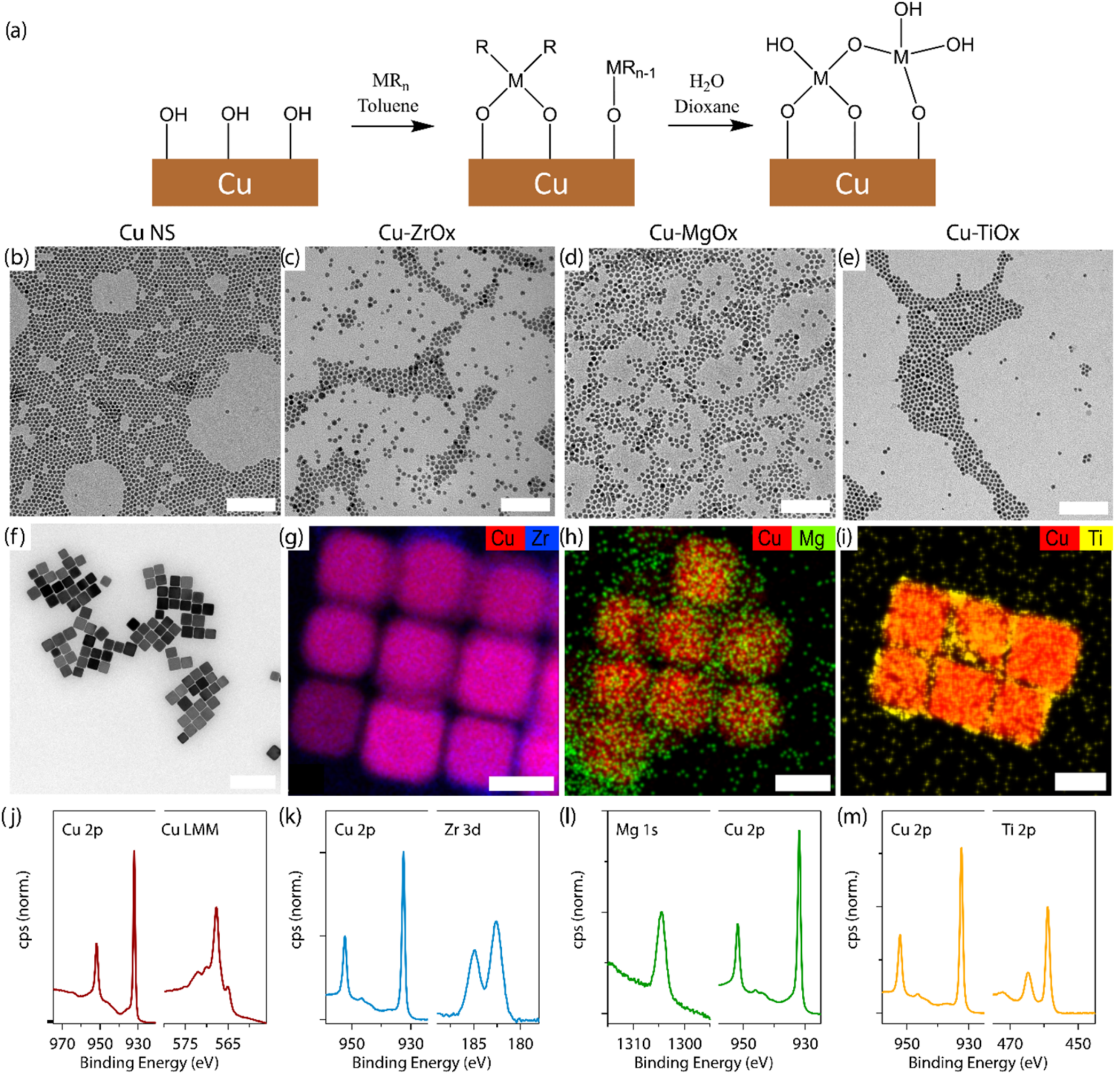
Chemistry and characterization of the Cu-MOx core–shell nanoparticles obtained by c-ALD. (a) Synthesis schematic of the stepwise formation of surface metal and oxygen bonds inspired by ALD. (b–e) TEM images of (b) Cu NS, (c) Cu-ZrOx, (d) Cu-MgOx, and (e) Cu-TiOx. Scale bar = 100 nm. (f) Bright-field TEM images of Cu nanocubes. Scale bar = 200 nm. STEM-EDX maps of (g) Cu-ZrOx nanocubes, (h) Cu-MgOx nanocubes, and (i) Cu-TiOx nanocubes. Scale bar = 30 nm. X-ray photoelectron spectroscopy spectra at the relevant core-level binding energy for (j) Cu, (k) Cu-ZrOx, (l) Cu-MgOx, and (m) Cu-TiOx NS.

A synthesis schematic is reported in Figure 1a. Inspired by the gas-phase ALD chemistry and considering the commercial availability of the precursors, we chose tetrakisdimethylamidotitanium, tetrakisdimethylamidozirconium, and cyclopentadienyl magnesium to deposit TiO_2_, ZrO_2_, and MgO on Cu nanocrystals, respectively.^54−56^ Carboxylic acid ligands and hydroxyl groups can serve as nucleating sites for the oxide.^17,48−51^ The as-prepared Cu nanocrystals are surface-functionalized with ligands (i.e., amines and phosphonates) which do not act as nucleating sites for the oxide shell; thus, we proceeded with preoxidizing the surface of the Cu nanocrystals with H_2_O_2_ to introduce hydroxyl groups.^17,48−51^ The organometallic precursors are then titrated in a dispersion of the Cu nanocrystals in dry apolar solvent and are driven to react on the surface with these hydroxyl groups. We then used stoichiometric amounts of water to convert the surface-anchored precursors into an oxide matrix. In previous work, we used iso-propanol as co-reactant.^17^ Here, we find the higher reactivity of water as a co-reactant to be crucial to the process because not all of the metal oxide precursors decompose to the corresponding oxide from the respective metal isopropoxides (Figure S1). In order to use water, we switched to dioxane as the solvent because dioxane serves as a good carrier for water to organic solvents. A surface titration of the hydroxyl groups with the organometallic precursors allows to avoid unreacted precursor in solution during the nucleation step. We found a 2:1 stoichiometric ratio of water to the metal–organic precursor during the growth to be optimal for the formation of the oxide coating while avoiding loss of colloidal stability of the Cu nanocrystals (Figure S2). We targeted a composition of 30% metal oxide (M/M+Cu), which corresponds to an at most 1–2 nm thin surface layer. This thickness falls in the optimal thickness range for CO_2_RR based on our previous study on similar nanostructures with aluminum oxide (Figure S3).^17^

We applied this chemistry to both Cu nanospheres (NSs) and nanocubes (Figure 1b–i). The higher surface-to-volume ratio of the Cu nanospheres facilitates the data interpretation from surface characterization techniques. The bigger size and extended surfaces of the Cu nanocubes ease the characterization via microscopy techniques. A representative transmission electron microscopy (TEM) image of the Cu NS starting material is presented in Figure 1b, showing a Cu NS of approximately 7 nm in diameter. The quasi-spherical particle shape is maintained during c-ALD, yielding the isolated and purified particles for Cu-ZrOx, Cu-MgOx, and Cu-TiOx (Figure 1c–e). TEM images of physical mixtures prepared as reference materials are presented in the Supporting Information, showing large micrometer-sized networks of metal oxide, in stark contrast to the samples where metal oxide is promoted to grow on the nanospheres (Figure S4). The coating of the oxide on the Cu nanocrystals becomes more evident in scanning transmission electron microscopy–energy-dispersive X-ray spectroscopy maps (STEM-EDX) when the Cu nanocubes are used (Figures 1f–i and S5). Finally, the X-ray photoelectron spectroscopy (XPS) on purified dispersions of the colloidal synthesis product of Cu NS, Cu-ZrOx, Cu-MgOx, and Cu-TiOx is reported in Figure 1j–m. The respective spectra demonstrate clear signals for Cu 2p, Zr 3d, Mg 1s, and Ti 2p photoelectrons. The binding energies for Zr 3d_5/2_ (182.6 eV), Mg 1s (1304.4), and Ti 2p_3/2_ (458.9 eV) correspond to tabulated values of the corresponding oxides ZrO_2_, MgO, and TiO_2_, confirming the successful shell growth of metal oxides on Cu. Based on this characterization, we refer to the synthesized nanomaterials as Cu-MOx core–shell nanoparticles, so those including the Cu NSs are Cu-MOx core–shell NSs.

### Electrocatalytic Testing

After successfully expanding the library of tailored Cu-MOx nanostructures accessible in the form of core–shell with extensive interfacial contacts between the Cu and the MOx components, we focused on those including the Cu nanospheres for the study of their electrocatalytic properties. Indeed, Cu NS are widely studied in the literature for the reduction of CO_2_ and provide a good benchmark system due to their structural instability at cathodic bias, facile synthesis, and known product distribution tested by several groups.^17,57^

**Figure 2:**
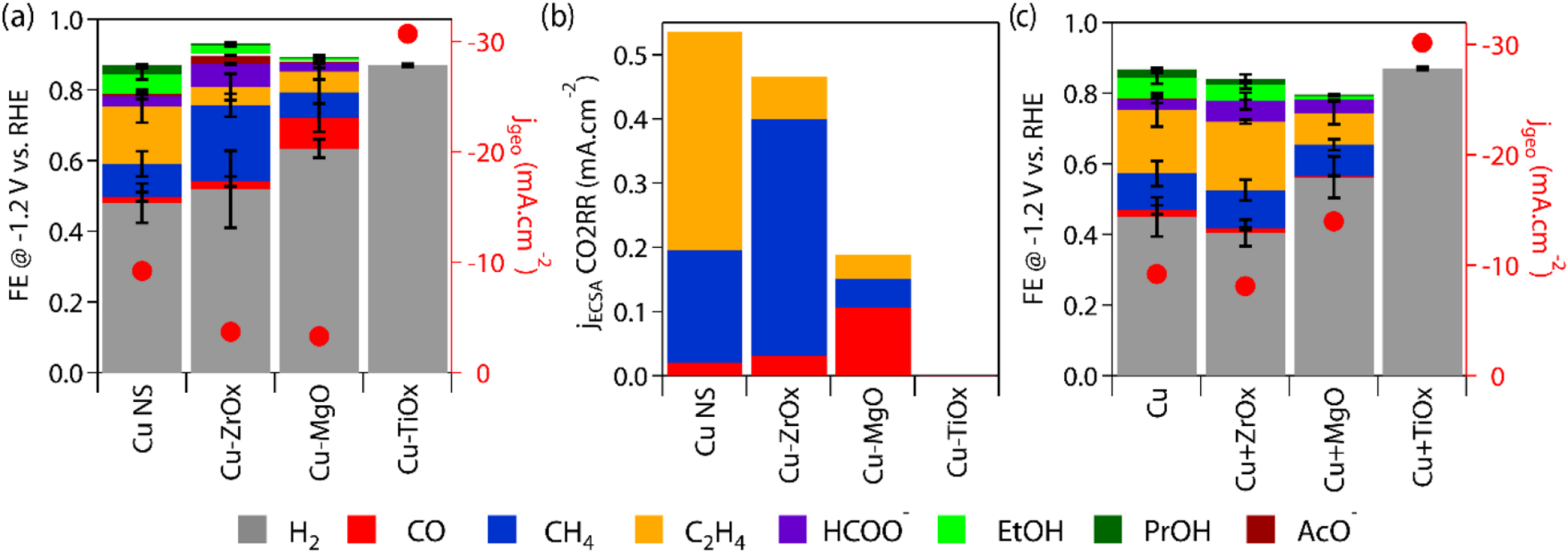
CO_2_RR electrocatalytic performance of core–shell and physical mixtures of Cu and MOx at −1.2 V vs RHE. (a) Faradaic efficiency and cathodic current of core–shell Cu-MOx NSs. (b) Product-specific partial current density normalized by electrochemically active surface area for the core–shell Cu-MOx NSs. (c) Faradaic efficiency and cathodic current of physical mixtures of equal composition. Color code for the products applies to all panels. The measurements are averaged data collected over 1 h of chronoamperometry in an H-cell with a 0.1 M KHCO_3_ electrolyte.

We compared the behavior of the Cu-MOx NSs to that of the corresponding physical mixtures to investigate the importance of shared interfaces. The electrocatalytic performance of the Cu-MOx and the physical mixtures of equal stoichiometry are presented in Figure 2. We identified −1.2 V vs RHE (reversible hydrogen electrode) as the most relevant potential to compare the catalysts based on different screenings (Figure S7). Similar trends were also seen at −1.1 V vs RHE (Figure S8).

Interestingly, interfacing Cu NSs with ZrOx, MgOx, or TiOx yields a shift of selectivity toward methane, CO, and promotion of the competing hydrogen evolution reaction (HER), respectively (Figure 2a). Concomitantly, Cu-ZrOx and Cu-MgOx display a suppressed cathodic current, while Cu-TiOx shows an increased current (Figure 2a). The values of the CO_2_RR current density normalized by the electrochemically active surface area (*j*_ECSA_) of the catalysts indicate that the intrinsic activity follows a similar trend in the promotion of methane, CO, and HER for Cu-ZrOx, Cu-MgOx, and Cu-TiOx (Figure 2b). Interestingly, the *j*_ECSA_ values for CO_2_RR of Cu and Cu-ZrOx are similar, which indicates that the intrinsic activity for CO_2_RR of the two catalysts is similar. As both *j*_ECSA_ for methane and hydrogen is greater in Cu-ZrOx than Cu (Table S1), the near-interface sites most likely facilitate the adsorption of protons, favoring both HER and CH_4_ pathways on the accessible Cu sites of the core–shell catalyst.^17^ On the contrary, the intrinsic CO_2_RR activity of Cu-MgOx and Cu-TiOx drops.

Moving to the physical mixtures (Figure 2c), neither ZrOx nor MgOx have any major effect on the CO_2_RR product distribution or the current density of the Cu NSs. Instead, the physical mixture Cu + TiOx has a behavior similar to Cu-TiOx, which is promoted by HER and increased current density.

### Investigation of Electronic Effects Arising from Cu|MOx Interfaces

Generally, Cu speciation remains an important catalyst characteristic driving CO_2_RR selectivity in different systems, including in doped Cu,^58^ copper oxide derived Cu,^59,60^ as well as Cu electrocatalysts under pulsed operation.^29,61,62^ The catalytic behavior of copper materials interfaced with metal oxides for CO_2_RR has been often connected to electronic effects arising at the interface from the stabilization of oxidic Cu species.^17,20,30,31,34,63^ Furthermore, the aforementioned study on Cu-AlOx core–shell NSs has correlated the increased stabilization of oxidic Cu species at the Cu|AlOx interface to a promoted production of methane and a suppression of Cu restructuring.^17^

**Figure 3:**
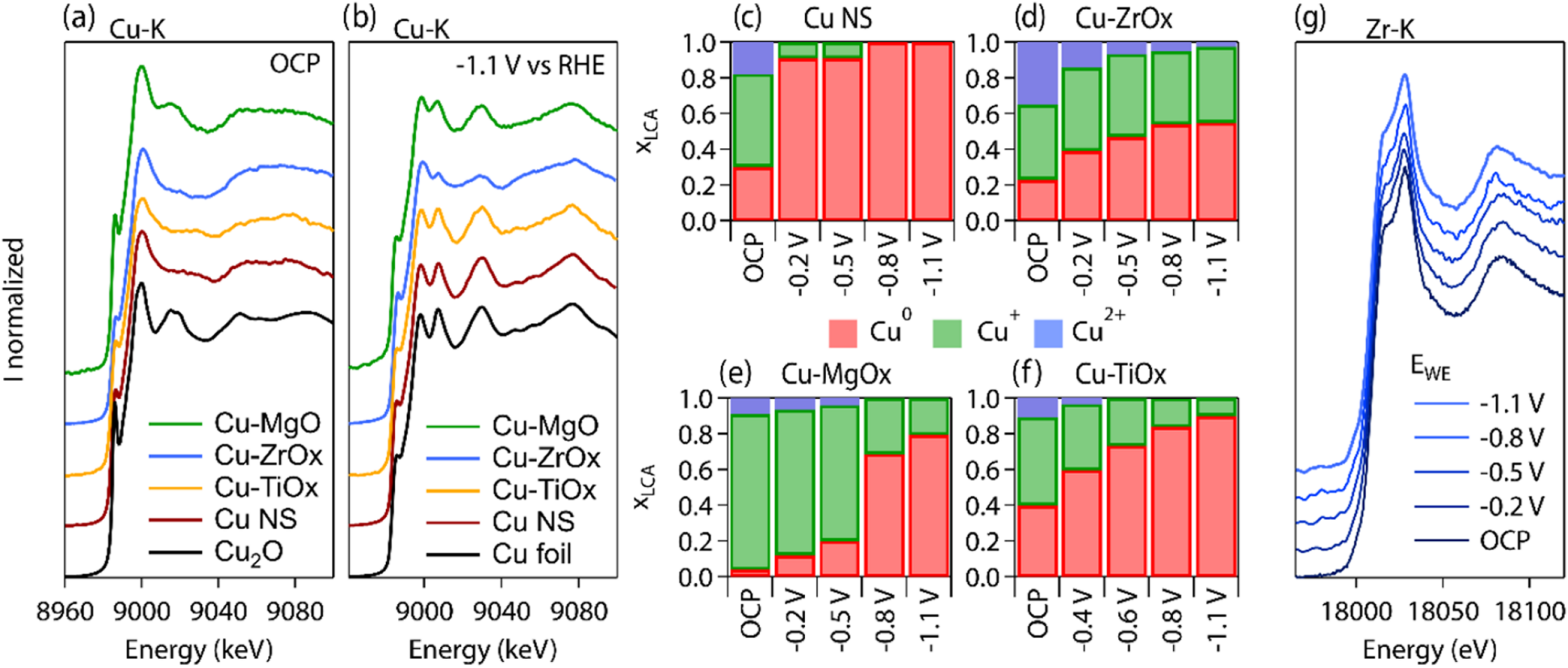
Operando X-ray absorption spectroscopy on Cu and Cu-MOx catalysts to monitor interfacial electronic effects and copper speciation. Spectra in the near-edge region for tested electrocatalysts at (a) open-circuit potential and (b) −1.1 V during CO_2_RR. (c–f) Cu speciation as determined by linear combination analysis at consecutively more negative potentials for (c) Cu NSs, (d) Cu-ZrOx, (e) Cu-MgOx, and (f) Cu-TiOx. (g) Evolution of the X-ray absorption near-edge spectrum at the Zr K-edge for Cu-ZrOx during CO_2_RR.

To correlate the observed electrocatalytic performance of the synthesized materials to electronic interfacial effects and copper speciation, we turned to operando X-ray absorption spectroscopy (XAS) and, more specifically, X-ray absorption near-edge spectroscopy (XANES) (Figures 3 and S17–S22).

The Cu K-edge XANES data of all samples, which are Cu and Cu-MOx, present a strong characteristic pre-edge feature at 8986 eV, which corresponds to Cu_2_O, at open-circuit potential (Figure 3a). The characteristic metallic features of Cu at 9007 and 9030 eV are identified in the spectra of all samples after 1 h at the operating CO_2_RR potential (Figure 3b). Noticeably, the metallic features are less pronounced for Cu-ZrOx compared to the other samples, which hints at preserved oxidic Cu at strongly cathodic bias.

To quantify the speciation of Cu at the tested potentials, molar fractions *x*_LCA_ obtained by linear combination analysis (LCA) with standard XANES spectra are presented for Cu NS, Cu-ZrOx, Cu-MgOx, and Cu-TiOx vs the working electrode potential (Figure 3c–f). In line with the qualitative evolution of the spectra, the Cu NSs become entirely metallic already at −0.2 V vs RHE. In Cu-MgOx, the contribution of metallic Cu estimated by LCA fitting remains constant and below 25% until −0.8 V vs RHE, which then triggers a sudden reduction (Figures 3e and S21). In Cu-TiOx, the reduction of copper oxide to become predominantly metallic proceeds more gradually. In Cu-ZrOx, copper oxide is reduced as the potential becomes more negative, yet a substantial fraction of ∼40% of oxidic copper is still preserved at the most cathodic potential. The stability of oxidic Cu species in the Cu-ZrOx catalyst coincides with a remarkably stable Zr K-edge XANES spectrum as a function of potential (Figures 3g and S22). A reference experiment on the Cu + ZrOx physical mixtures indicates complete reduction to metallic Cu at −1.1 V vs RHE and a similarly stable Zr K-edge spectrum (Figures S22 and S23).

Altogether, these data indicate that all Cu|MOx interfaces stabilize the oxidized copper to some extent, which is also consistent with cyclic voltammetry experiments (Figure S11). However, the stability of the oxidic copper as a function of the potential and time depends on the chemical nature of the oxide.

If we look back at the product distribution, the results with ZrOx connect the stability of a significant fraction of the oxidic copper to the methane production, which is consistent with our work on Cu-AlOx NSs.^17^ There, the locked fraction of oxidized Cu was proposed to generate electronic effects that explain the observed methane selectivity; thus, we propose to extend a similar argument to the similarly stable Cu-ZrOx nanospheres. On the contrary, both Cu-TiOx and Cu-MgOx present a similarly smaller fraction of stable oxidic copper at the CO_2_RR cathodic potential, yet they differ in their catalytic behavior. Thus, catalyst features other than copper speciation must be considered to explain the reactivity.

### Correlating Catalyst Morphology with Cu|MOx Interface Stability

Curiously, although catalyst morphology, faceting, and Cu restructuring have been demonstrated to be crucial to the CO_2_RR product selectivity,^7,27,64−66^ morphological effects are mostly overlooked in Cu-metal oxide electrocatalyst studies. Despite the preservation of oxidic Cu hinting at the existence of a stable Cu|MOx interface,^17,20,23,36,67^ the link between the stability of the formed interfaces and the catalyst morphology remains unexplored.

**Figure 4:**
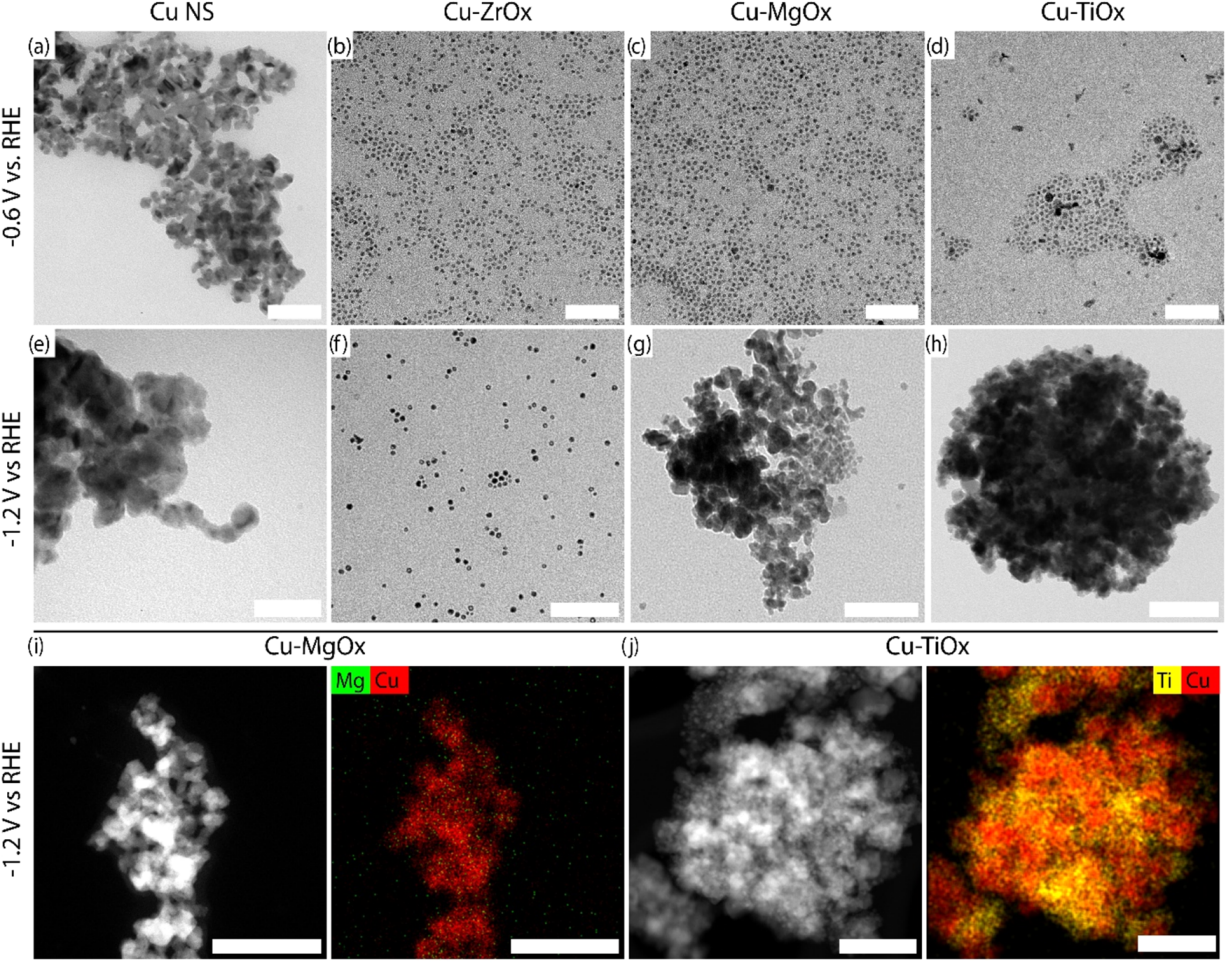
Post-CO_2_RR morphological and compositional characterization of core–shell Cu-MOx catalysts. (a–d) TEM images of (a) Cu NS, (b) Cu-MgO, (c) Cu-ZrOx, and (d) Cu-TiOx after 1 h of CO_2_RR at −0.6 V vs RHE. (e–h) TEM images of (e) Cu NS, (f) Cu-MgO, (g) Cu-ZrOx, and (h) Cu-TiOx after 1 h of CO_2_RR at −1.2 V vs RHE. Scale bars in (a–h) correspond to 100 nm. (i, j) HAADF-STEM images and the corresponding EDXS-color map of (i) Cu-MgOx and (j) Cu-TiOx after CO_2_RR at −1.2 V, indicating the disappearance of Mg from the electrode and the mixed phase solid formed during electrocatalysis with Cu-TiOx core–shell NSs. Scale bars correspond to 50 nm.

Thus, we utilized electron microscopy to assess the morphological stability of the synthesized core–shell structures and the Cu|MOx interfaces involved at different cathodic biases (Figure 4). In particular, we chose −0.6 V vs RHE, which is below the threshold voltage triggering Cu reduction in Cu|MgOx, and −1.2 V vs RHE, which corresponds to the CO_2_RR voltage.

TEM images indicate that the Cu NSs reconstruct dramatically already at a low cathodic bias (Figure 4a), which is consistent with literature reports on Cu NSs.^17,57,66,68−71^ Cu-ZrOx preserve their spherical morphology at both voltages, and operational stability is observed beyond the 1 h testing at −1.2 V vs RHE (Figure S10). Interestingly, the morphology of Cu-MgOx is stable at −0.6 V vs RHE, while some changes are observable in the morphology of a few Cu-TiOx NSs at the same voltage. Instead, Cu-MgOx and Cu-TiOx both drastically change their morphology at −1.2 V vs RHE. The same oxide-dependent restructuring is confirmed by changes in the estimated electrochemical surface area before and after electrochemical experiments (Figure S13). While Cu-MgOx and Cu-TiOx are similar in their reconstruction behavior at −1.2 V vs RHE from TEM images, high-angle annular dark-field scanning electron transmission microscopy (HAADF-STEM) with EDXS maps and ICP-OES quantification of the electrolyte indicate that Mg dissolves in the electrolyte, whereas TiOx remains on the electrode (Figures 4i,j and S6). The physical mixture of Cu and TiOx also yields a similarly reconstructed morphology postelectrolysis (Figure S16).

### Correlating the Nature of the Oxide, the Stability and the Reactivity of Cu|MOx Interfaces in CO_2_RR

**Figure 5:**
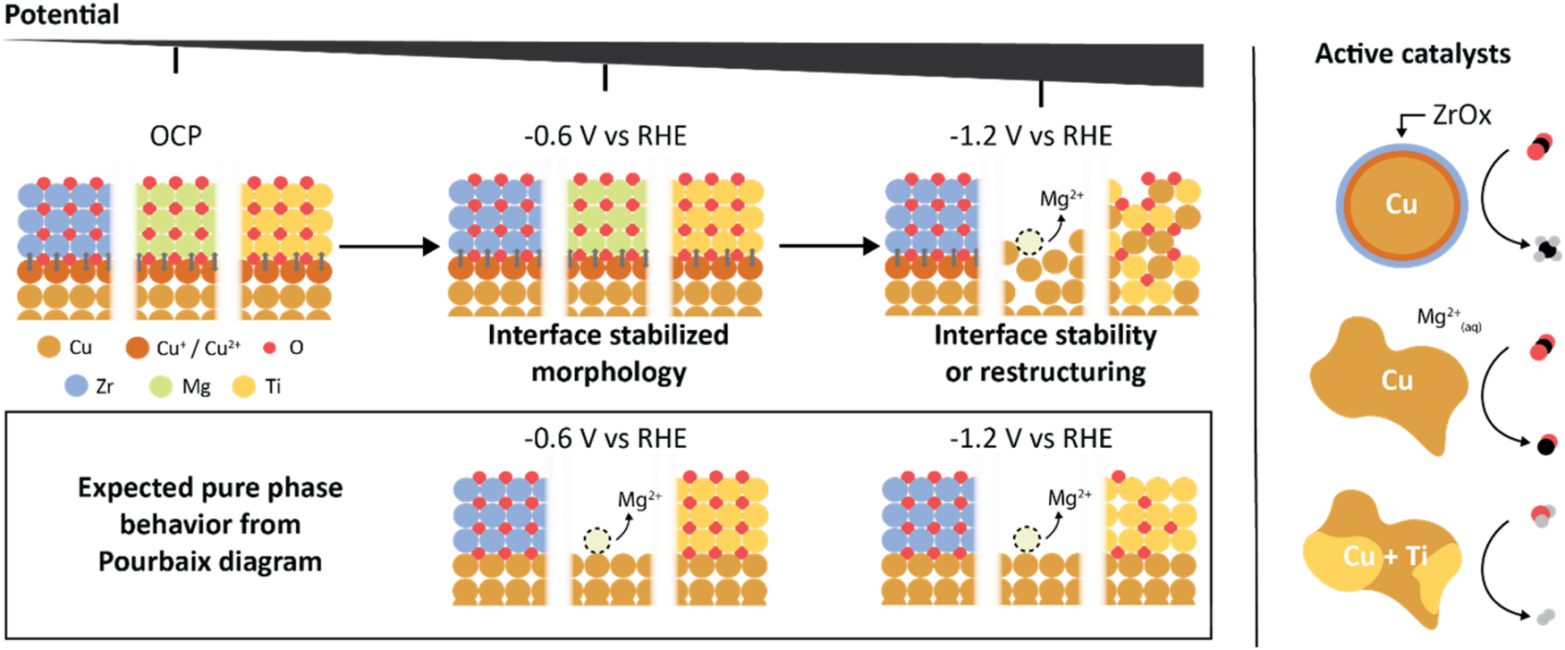
Rationalizing the behavior of Cu|MOx interfaces under cathodic potentials and in CO_2_RR. Although the Pourbaix diagram can provide insight into the possible fate of the respective materials, the presence of an interface changes the cathodic potential at which Cu is reduced and Mg ions dissolve and fall short in predicting the restructuring of the Cu-TiOx system. This work highlights that the bond strength at the interface has significant consequences for the fate of the constituent materials during electrochemistry and, thus, on the reactivity of the active catalysts.

At this point, an overall picture starts to shape up, which is summarized in Figure 5.

The ZrOx forms stable Cu–O–Zr interfacial bonds, which is reflected in both XAS and morphological stability of the catalysts. The Cu-MgO and the Cu-TiOx provide stable Cu–O–Mg and Cu–O–Ti along with a generally more stable catalyst morphology at lower voltages; however, these interfacial sites are eventually lost at the more cathodic potentials at which CO_2_RR occurs with Mg mostly dissolving and Ti remaining in some form mixed with Cu.

The stability of the Cu|ZrOx interface matches well with the Pourbaix diagram, where solid zirconium oxide remains stable until very cathodic potentials across a wide pH range.^43^ The product selectivity can be traced back to the stable interface, which locks the surface oxidic copper. The importance of the interface is also consistent with the fact that a Cu+ZrOx physical mixture does not generate any change in product distribution compared with Cu NSs. We hypothesize that the methane activity most likely arises from accessible near-interface sites.^17^ Thus, these oxidic sites are uniquely distinct from the oxidic copper sites identified in pulsed electrolysis experiments.^29,61,62,72^ Furthermore, the intrinsic activity for CO_2_RR being similar to pure Cu indicates that the created interfaces boost the reactivity of the near-interface copper sites accessible to CO_2_.^17^

The Pourbaix diagram indicates that magnesium oxide corrodes at any cathodic potentials and yields Mg^2+^ at pH < 10.^44^ Thus, the stability of Cu|MgOx interfaces up to at least −0.8 V vs RHE indicates that the Cu–O–Mg interfacial bonds stabilize the oxide against dissolution to a certain extent. Eventually, the cathodic potential reduces these bonds and dissolution occurs under CO_2_RR conditions. Regarding the CO selectivity, we exclude a major influence of the Mg^2+^ cations in solution because neither the Cu+MgOx physical mixture nor the addition of Mg^2+^ in the electrolyte promotes CO production (Figure S15). Thus, we propose that the initial presence of the interface impacts the reconstruction kinetics of Cu-MgOx compared to Cu NS and drives the formation of predominantly metallic, yet structurally different, Cu sites, which are selective for CO.

Finally, the Pourbaix diagram indicates that Ti^4+^ in TiO_2_ reduces to Ti^3+^ and no soluble species are expected at pH > 7.^45^ This behavior is similar to another reducible oxide, cerium oxide; yet Cu|CeOx interfaces remain stable under CO_2_RR conditions.^63^ A possible explanation for the gradual loss of stability in the Cu|TiOx interfaces might come from a voltage-driven chemical reactivity inducing a gradual change of composition and speciation.^73^ The postelectrolysis morphology and electrocatalytic behavior of physical mixtures are similar to the Cu-TiOx core–shell system, which is consistent with the idea of a voltage-induced chemical reaction independent of the existence of an interface.^73^ The observed increase in conductivity and HER selectivity could then be linked to the formation of highly active sites at the newly formed interfaces in the restructured Cu- and Ti-containing domains.^37^ However, future studies are needed to corroborate the operando speciation of Ti at these interfaces.

## Conclusions

In conclusion, this work proposes the chemistry to synthesize Cu-ZrOx, Cu-MgOx, and Cu-TiOx core–shell nanoparticles with comparable morphology, composition, and size. The proposed material chemistry is demonstrated to be versatile in both the composition of the shell oxides and the Cu nanoparticle core morphology. We then determined the behavior of the model system nanospheres under cathodic voltages with attention to both electronic and morphological characteristics during operation, which we eventually connect to the reactivity of these Cu-MOx materials toward CO_2_RR. We rationalize the impact of the chemical nature of the oxide on the stability and reactivity of the Cu|MOx interface by the electrochemical stability predicted by the Pourbaix diagram along with the oxide-specific Cu–O–M interfacial interactions.

These results highlight the importance of developing the chemistry of well-defined catalytic materials along with correlating the electronic properties of interfaces and the evolving catalyst morphology under operation to rationalize the performance differences. This study proves the importance of the morphological evolution in Cu-MOx CO_2_RR catalysts, which paves the way toward disentangling convoluted effects of catalyst composition, operando Cu speciation, and morphology on the ensuing CO_2_RR selectivity and stability. The lesson learned provides fundamental insight into shaping the next generation of Cu-MOx catalysts for selective and especially stable operation in the CO_2_RR and the same working framework is applicable beyond this reaction.

## Data Availability

Experimental data are openly available in Zenodo at doi.org/10.5281/zenodo.14980120.

## Supporting Information Available

Additional experimental details with regard to synthesis, characterization, and electrocatalytic testing, further catalytic testing results of Cu-MOx catalysts, as well as a thorough description of the X-ray absorption spectroscopy analysis (PDF)
