# Supplementary material for: The Chemical Nature of the Oxide Directs the Stability and Reactivity of Copper|Oxide Interfaces in the Electrochemical CO2 Reduction Reaction
Source: Chem Mater. 2025 Apr 24;37(9):3343–52. doi: 10.1021/acs.chemmater.5c00135 (PMC12079792; doi:10.1021/acs.chemmater.5c00135)
Supplement: Supplementary file 1 — cm5c00135_si_001.pdf [file cm5c00135_si_001.pdf]

Supporting Information

The chemical nature of the oxide directs the stability and reactivity of copper|oxide interfaces in the electrochemical CO_2 reduction reaction

Jari Leemans[1,#], Jennifer Calderon Mora[1,#], Petru P. Albertini[1], Krishna Kumar[1], Coline M.A. Boulanger[1], Raffaella Buonsanti[1,*]

1: Laboratory of Nanochemistry for Energy, Institute of Chemical Sciences and Engineering, École Polytechnique Fédérale de Lausanne, Sion, Switzerland

Author Contributions: J.L. and J.C.M. contributed equally.

Additional Experimental Information

Synthesis and surface oxidation of 7 nm Cu nanocrystals

20 mL of trioctylamine is degassed under vacuum for 1 hour at 120°C connected to a Schlenk line in a three-neck flask equipped with stirring bar and heating mantle. After degassing, the flask is filled with N2 and cooled to 50°C. The septum is opened and 272 mg (1 mmol) tetradecylphosphonic acid and 242 mg (2 mmol) Cu(I)OAc are added to the three-neck flask. The reaction mixture is heated to 180°C under N_2. After 30 minutes at 180°C the temperature is increased to 270°C. After 30 minutes at 270°C the reaction mixture is cooled by removing the heating mantle. The deep red dispersion is transferred air-free to an inert atmosphere glovebox. The Cu nanocrystals are isolated by three consecutive cycles of precipitation/redispersion with toluene and ethanol. The final pellet is dispersed in 5 mL of toluene to yield a concentrated stock solution.

To prepare the Cu nanocrystals for c-ALD, the dispersed nanospheres are titrated with a surface-stoichiometric amount of a 0.05 M hydrogen peroxide solution in EtOH, followed by precipitation and redispersion in dry toluene. The deliberate oxidation of the surface in toluene was deemed essential for the nucleation of the oxide shell on Cu.[1]

Synthesis of 40 nm Cu cubes

Tri-n-octylphosphine oxide (24 mmol, 9.37 g) was first degassed in a 250 ml three-neck flask equipped with reflux condenser and internal thermocouple temperature controller and degassed under vacuum with vigorous magnetic stirring at room temperature. Then, oleylamine (116 mL) was added to the flask and once again degassed for 1 hour at 130 °C. After the solution was allow to cool down to room temperature, CuBr (5 mmol, 0.71 g) was quickly added to the solution under nitrogen flow. The temperature is first raised to 80°C and maintained for 15 minutes, then is quickly raised to 260°C and kept for one hour. The solution is then allowed to cool down to room temperature and transferred to a glove box. The Cu cube dispersion is isolated in three cycles of precipitation/redispersion in toluene/EtOH. Tri-n-octylamine (100 μL) was added during the washing steps to help maintain the colloidal stability. The precipitate was finally recovered with toluene and stored in a glove box.

Nuclear magnetic resonance spectroscopy

1D 1H-nuclear magnetic resonance spectra were recorded on a Bruker Avance IIIHD-400 spectrometer equipped with a $BBFO_2$ probe. Quantitative spectra were recorded with 90 degree pulsing and 45 second waiting times between spectra to ensure complete relaxation. All spectra are recorded in deuterated toluene.

Inductively coupled plasma optical emission spectroscopy

Quantification of Cu, Mg, Zr and Ti concentration was performed by inductively coupled plasma optical emission spectroscopy on an Agilent ICP-OES 5110 instrument. Colloidal dispersions were sampled, the dispersing solvent was dried under N_2 flow and the remaining powder was consequently digested in concentrated nitric acid. After dilution the samples as well as a series of calibration standards prepared from standard reference solutions of each element were injected into the instrument. Concentrations were determined from the ensuing calibration curve.

Supplementary Data

S1 Synthesis and Additional Characterization

S1.1 Establishing the reaction chemistry for colloidal metal oxide growth

c-ALD requires a reactive metal-organic precursor and an oxygen source to grow the respective oxide on the colloidal nanocrystal core of choice. The first c-ALD oxygen source was molecular oxygen to grow alumina in combination with tri-methylaluminium.[2,3] When applying this same synthesis scheme to Cu nanocrystals, oxygen caused undesired oxidation of the metallic core.[1] As a replacement for oxygen, isopropanol was introduced as a mild oxygen source to react with trimethylaluminium.[1] Exploring the isopropanol route in this study we quickly identified that the addition of stoichiometric amounts of isopropanol to tetrakisdimethylamidozirconium would yield the formation of a stable zirconium isopropoxide in toluene, which does not decompose to yield the oxide at mild reaction conditions studied in this work. The formation of Zr-isopropoxide is identified from the characteristic chemical shift of protons in the alpha position to the alkoxide moiety in H-NMR (**Figure S1)**. No precipitate indicative of the metal oxide appears when titrating isopropanol to TDMA-Zr.

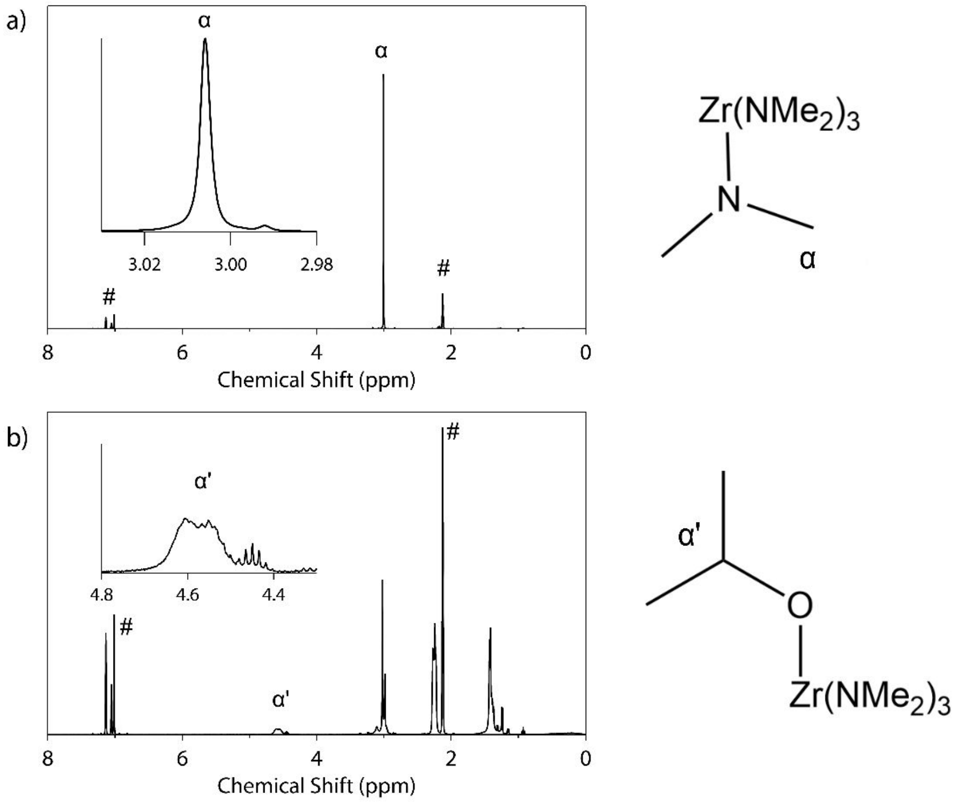

Figure S1: a) ^{1}H-NMR spectrum of TDMA-Zr in toluene-d8. The single resonance annoted as α corresponds to the methyl protons in dimethylamide. # denotes solvent protons. b) 1H-NMR spectrum of the same solution after addition of 1 equivalent of isopropanol per Zr atom. The α' resonance corresponds to isopropoxide protons in the α-position to the alkoxide group. Multiple substitutions on a single metal centre explain the heterogeneity in the resonance.

The decision to use water in dioxane as an oxidizing agent was made because isopropanol proved ineffective at transforming the selected precursors in this study. Titrating a dilute solution of water in dioxane to an equimolar solution of the three metal-organic precursors (TDMA-Zr, TDMA-Ti, $MgCp_2$) in toluene immediately forms a precipitate which we identified to be the respective oxides. With the use of ^{1}H-NMR we identified a 2:1 stoichiometry between water and the metal-organic precursors to be ideal to fully convert the respective precursors (**Figure S2**).

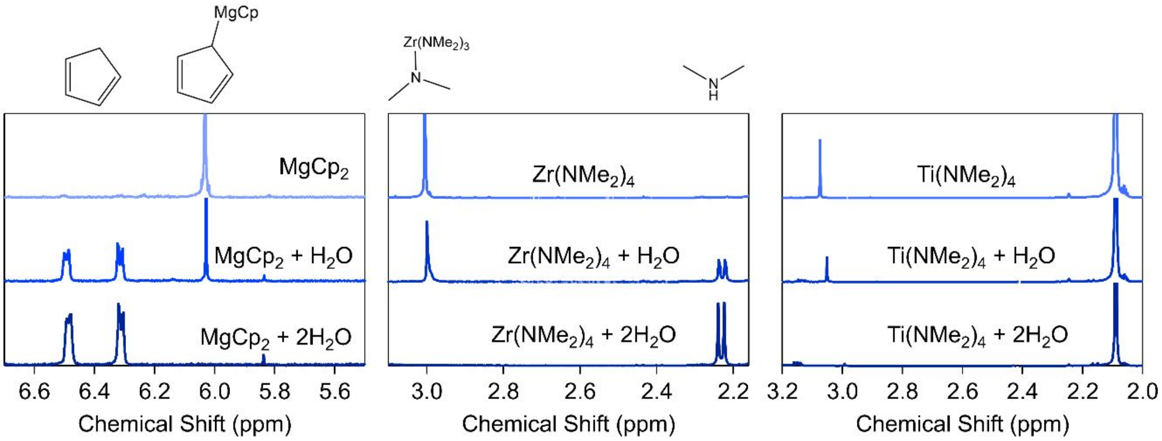

Figure S2: Stepwise titration of the metal organic precursors $MgCp_2$, TDMA-Zr and TDMA-Ti with dilute solutions of water in dioxane indicated a 2-fold excess of water is necessary to fully oxidize the precursors, as demonstrated by the appearance of the protonated ligands and disappearance of the initial resonance of the pure complex in toluene-d8. A yellow/brown precipitate is formed in the case of $MgCp_2$, and a white precipitate in the case of TDMA-Zr and TDMA-Ti.

S1.2 Calculating the expected shell size for different ratios of titrated metal and Cu

We aimed at 2 nm thickness as an upper limit for the thickness of the shell, based on our previous work on AlOx.[1] From the molar volumes $V_{m,i}$ of MgO, ZrO_2, TiO_2 and Cu, an estimate can be made on the required mole fraction x_i of titrated metal M in the final structures to attain the desired total nanocrystal size (diameter *d*) and shell thickness (**Figure S3**). The respective calculation follows the equations below:

$$\frac{n_M}{n_{Cu}} = \frac{V_{shell}}{V_{core}}\frac{V_{m,Cu}}{V_{m,MOx}} = \frac{V_{final} - V_{core}}{V_{core}}\frac{V_{m,Cu}}{V_{m,MOx}}$$

$$x_M = \frac{V_{shell}/V_{m,MOx}}{V_{shell}/V_{m,MOx} + V_{core}/V_{m,Cu}}$$

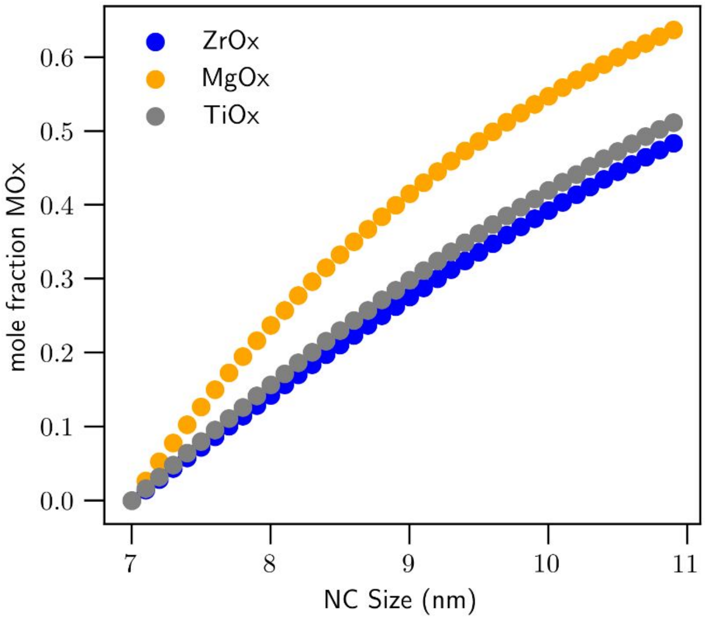

Figure S3: The maximum expected total NC size after shell growth on 7 nm spheres can be estimated from the molar volumes of the respective oxides and the mole fraction of Cu and metal oxide. With a mole fraction of 0.3 (M/M+Cu), the shell thickness would be at most 2 nm for the respective oxides, assuming a 100% chemical yield and conformal growth.

S1.3 Physical mixtures of Cu nanospheres and metal oxide

To compare the performance of the core-shell structures in the main manuscript to electrodes of equal composition but without explicitly introduced interfaces between metal oxide and Cu, physical mixtures were prepared. Physical mixtures are prepared from the same starting materials, but the precursors for the shell are reacted to form the oxide before the Cu nanospheres are added. The total composition of the sample yields a same metal oxide mole fraction of 0.3, as in the core-shell structures. All metal oxides prepared in this way resemble each other in morphology, yielding network-like structures interspersed with the well-defined Cu nanospheres (**Figure S4**).

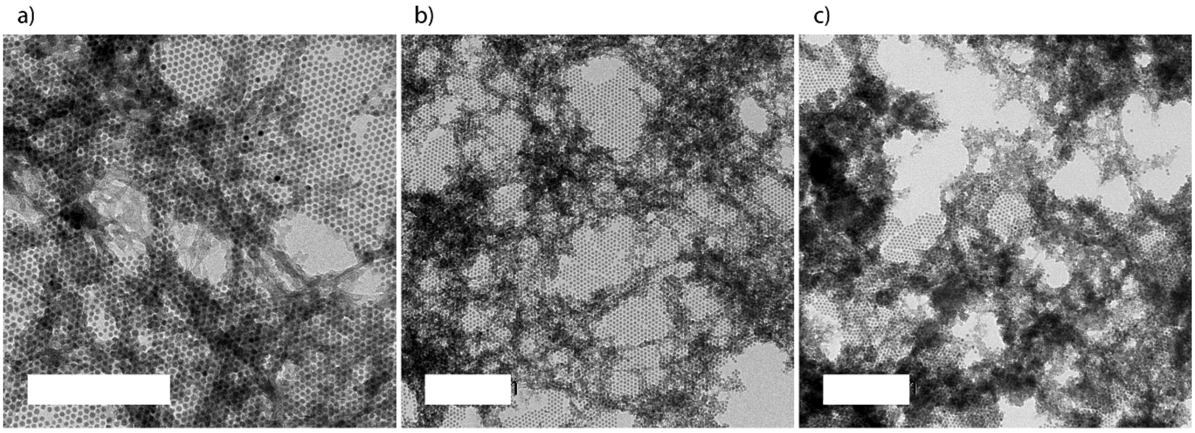

Figure S4: Bright-field transmission electron microscopy images of physical mixtures of Cu nanospheres and a) MgO, b) ZrO2, and c) TiO2. The metal oxides are synthesized by titrating the same water in dioxane solution to solutions of MgCp2, TDMA-Zr and TDMA-Ti in toluene, respectively. Scale bars are 200 nm.

S1.4 Energy dispersive x-ray spectra of the elemental maps in the main manuscript

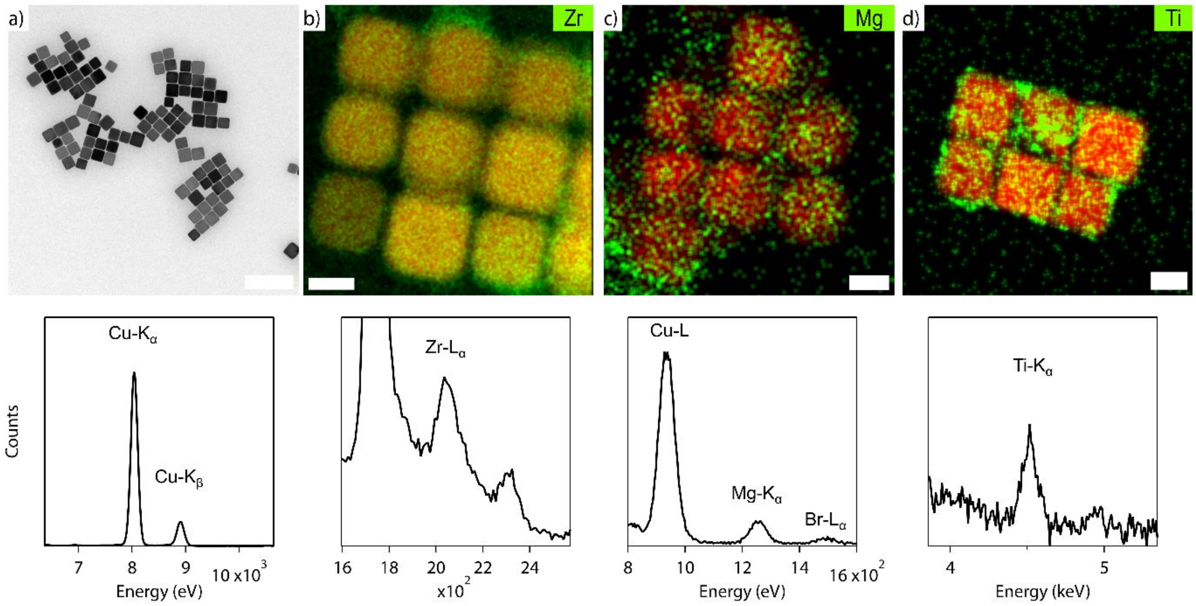

Figure S5: Energy-dispersive x-ray spectroscopy spectra are provided below the respective elemental maps, indicating well-resolved spectral lines corresponding to the elements in the metal oxide shell. Scale bars correspond to a) 200, b-c) 20 nm.

S1.5 Solubility of the metal oxides in 0.1 M $KHCO_3$ electrolyte

As discussed in the main text, the Pourbaix diagram of the respective oxides predicts the electrochemical stability for ZrO_2, a reduction to a solid Ti_2O_3 phase for TiO_2 and solvated Mg^{2+} ions for MgO in the potential-pH window of our CO2RR experiments. To verify the predicted behavior, the oxides were prepared from the ALD precursors by titrating a 40 mM water in dioxane solution to a 40 mM precursor solution in toluene. The precipitate was isolated by centrifugation and decantation. 1 mL of 0.1 M $KHCO_3$ electrolyte was added to the precipitate and centrifuged for 1 hour. The solutions were sampled with a micropipette, diluted in nitric acid and the metal presence was quantified with ICP-OES. No titanium was found in the solution, the sample yielded a small signal for Zr in the solution which only varied by a factor 2 compared to the blank signal, and a high concentration of Mg was found. The experiment proves that the bare MgO dissolves completely in our electrolyte, whereas for Ti and Zr we can exclude dissolution as a main degradation pathway. This result coincides with x-ray absorption spectroscopy data on the stability of our ZrOx phase during electrochemistry.

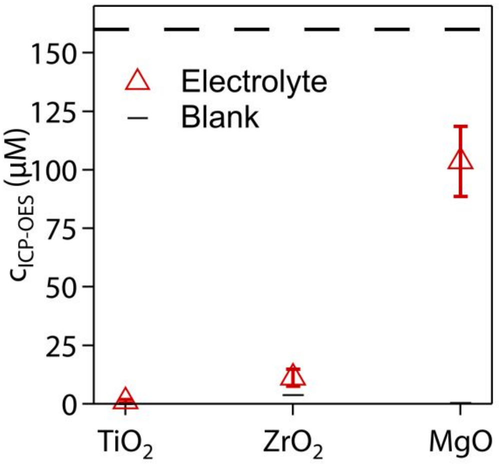

Figure S6: Concentration of Ti, Zr and Mg found in the electrolyte after 1 hour of dissolution in $KHCO_3$ electrolyte.

S2 Additional Electrochemistry Data

S2.1 Potential dependent CO2RR

To identify at which potential the model systems showed the clearest differences in selectivity and activity, 40 minutes of chronoamperometry were performed at potentials between -0.7 and -1.4 V vs RHE on Cu and Cu-ZrOx nanospheres (**Figure S7**). Selectivity and differences in CO2RR current are visible at all studied potentials, but the most drastic differences are obtained at -1.2 V vs RHE and beyond. Consequently, the data at -1.2 V vs. RHE was chosen to be presented in the main manuscript.

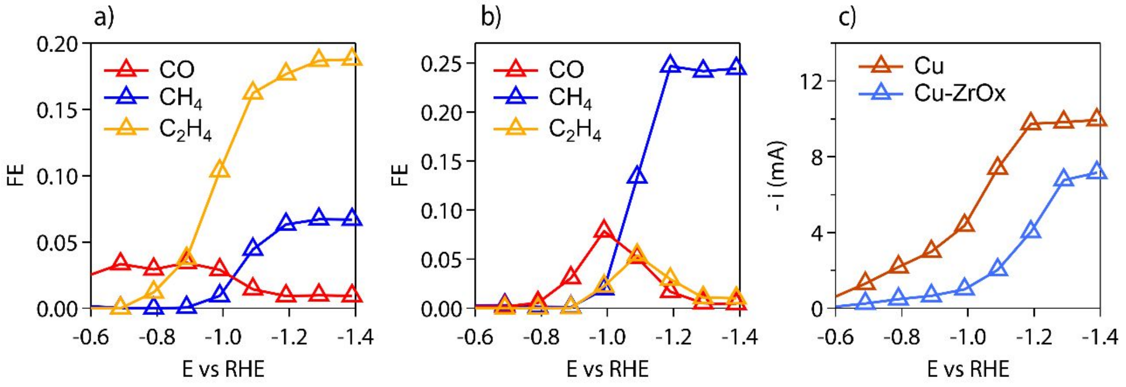

Figure S7: Potential dependent faradaic efficiency of gas products from the CO2RR on a) Cu nanospheres and b) Cu-ZrOx core-shell nanospheres. c) Cathodic current at the respective potentials for both samples.

S2.2 CO2RR Electrocatalysis at -1.1 V vs RHE

To supplement the data from the main manuscript, the individual core-shell samples were measured also at -1.1 V vs RHE. The trends in the data are similar, with a promoted selectivity for methane, CO and the hydrogen evolution reaction of Cu-ZrOx, Cu-MgOx and Cu-TiOx (**Figure S8**).

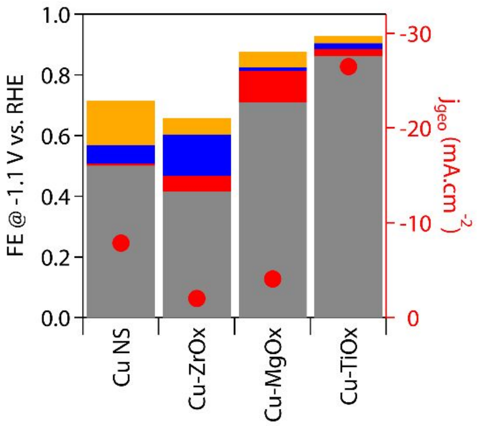

Figure S8: Faradaic efficiency of gas products and cathodic current for the bare Cu NS and the 3 core-shell model systems.

S2.3 Blank tests: CO2RR on pure oxides without Cu

To exclude any possible contributions from tandem catalysis or the activity of the pure oxides, CO2RR at -1.2 V was tested over the bare metal oxides without addition of Cu (**Figure S9**). The metal oxides are prepared from the same precursors used in c-ALD shell growth and the preparation of the physical mixtures. 15 micrograms of the respective metal oxide was dropcasted on the electrode to emulate the sample preparation of our Cu electrodes. None of the oxides yields current greater than obtained over bare glassy carbon substrates, and only in the case of TiOx is a small amount of CO detected. The low currents allow us to exclude any significant contribution of reactions occurring on the metal oxide surfaces, and the catalytic effects discussed in the main manuscript arise from the altered electronic properties and morphological evolution of the Cu surface.

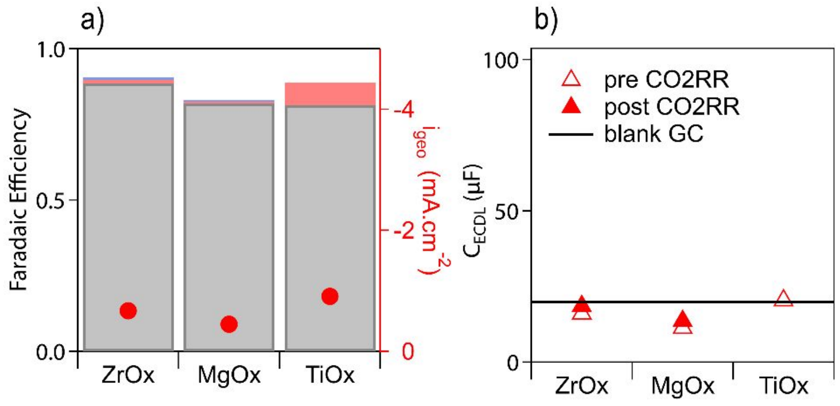

Figure S9: a) Faradaic efficiency and cathodic current obtained during 1 hour of electrolysis on the pure oxides deposited on glassy carbon. (grey = H_2, red = CO, blue = CH_4) b) The double layer capacitance estimated from cyclic voltammetry lies close to the value obtained on the bare glassy carbon (GC) substrates, suggesting the electrochemical surface area is unaffected by the presence of the network like structure on the flat glassy carbon electrodes.

S2.4 Catalytic stability of Cu-ZrOx and Cu-MgOx

To obtain insight into the catalytic stability of Cu-ZrOx and Cu-MgO core-shell systems, 14 hours of CO_2RR were performed over both structures at -1.2 V vs RHE (**Figure S10**). The gas products sampled are averaged per 30 minutes and plotted alongside the cathodic current. We associate the increase of ethylene after several hours of operation in Cu-ZrOx to restructuring based on previous work in our group and across the literature.[4–6] Such increase of ethylene occurs during the start-up phase for the bare Cu NSs and only after 6 hours in the case of Cu-ZrOx NSs, which indicates the superior stability of the latter. Previous work with CuAlOx suggests that further optimization of the system (e.g. shell thickness and/or porosity) might enable a further enhancement of the stability.[1]

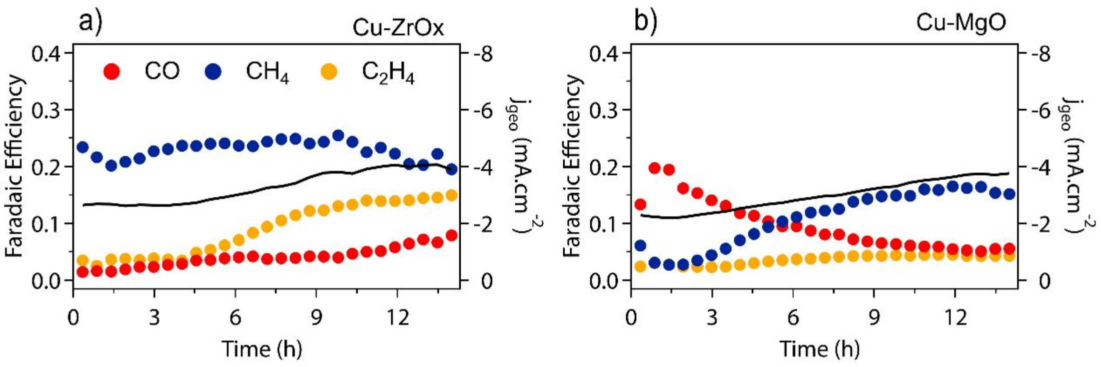

Figure S10: Catalytic stability of the a) Cu-ZrOx and b) Cu-MgOx nanospheres. The methane selectivity in Cu-ZrOx arising from the stable interface persists over the 14 hour experiment, although ethylene starts to appear as a by-product. In Cu-MgOx, the CO selectivity is lost and replaced by methane as the main gas product formed.

S2.5 Cyclic voltammetry

To corroborate the suppression of Cu redox transitions in the Cu-MOx core-shell system identified with operando x-ray absorption spectroscopy, we probed the same Cu speciation with cyclic voltammetry (**Figure S11**). Electrodes were prepared with the same 15 microgram of Cu mass loading used for CO2RR. For pure Cu (**Fig S11 a**), typical oxidation and reduction transitions can be seen around 0 V and -0.1, -0.2 and -0.4 V vs Ag/AgCl respectively.[1] These same transitions are heavily suppressed in all core-shell systems, as can be deduced by the total oxidizing and reducing current in the same samples (**Figure S11b, c and d**). At low potentials, all metal oxides suppress the ability of Cu to reduce at cathodic and oxidize at anodic bias. As discussed in the main manuscript, the stability only extends to CO2RR potentials for Cu-ZrOx.

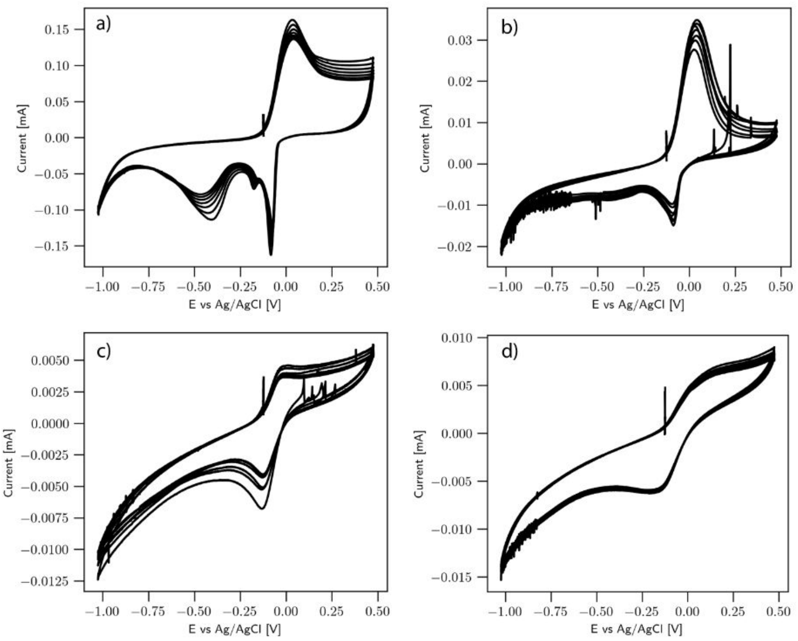

Figure S11: Consecutive cycles of cyclic voltammetry on a) Cu NS, b) Cu-ZrOx, c) Cu-MgOx and d) Cu-TiOx.

S2.6 Loss of stability probed via cyclic voltammetry

To corroborate the suppression of the redox transitions to the morphological evolution at cathodic bias, we performed an additional experiment where the cyclic voltammogram on Cu-TiOx was recorded before and after a 400 second chronoamperometry experiment at -1.2 V vs RHE (**Figure S12**). The initial voltammogram demonstrates that Cu redox transitions are heavily suppressed. At -1.2 V versus RHE, the cathodic current quickly increases towards the values reported in the main manuscript (**Figure S12b)**. After the noted increase in current, where the electron microscopy images demonstrate a strong restructuring of the core-shell catalysts, the voltammogram indicates that Cu is now readily oxidized and reduced during voltammetry (**Figure S12c**). The sequence of electrochemical experiments confirms that the TiOx shell is only able to stabilize the Cu surface in a limited potential window, whereas under CO2RR conditions the interface is ruptured and Cu once again readily undergoes potential induced redox chemistry.

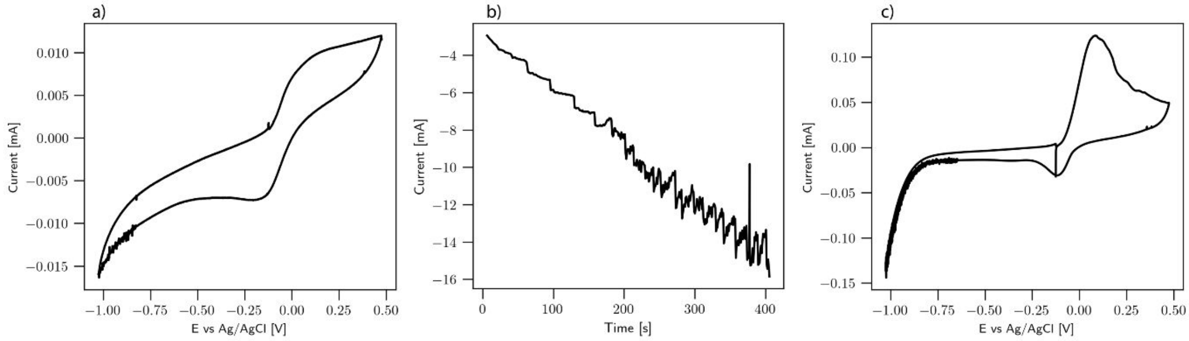

Figure S12: a) Cyclic voltammetry of the as-prepared Cu-TiOx on glassy carbon electrode. b) Current during 400 seconds of chronoamperometry at -1.2 V vs RHE. c) CV after the 400 second chronoamperometry experiment. The recovery of the Cu redox transitions after the large cathodic bias coincides with the drastic restructuring of Cu-TiOx at CO2RR potentials in the main manuscript and reiterates the limited potential window in which TiOx is able to stabilize the Cu surface.

S2.7 Electrochemical surface area evolution during electrochemistry experiments

ECSA data collected before and after electrochemistry show that the ECSA of the Cu NSs and of Cu-ZrOx does not change during CO2RR. Instead, the ECSA of Cu-MgOx and Cu-TiOx increases post-electrolysis (**Figure S13a**). This finding coincides with the morphological stability observed for the Cu-ZrOx nanospheres, and the restructuring observed in Cu-TiOx and Cu-MgOx. The ECSA of bare Cu does not change during electrochemistry because the restructuring occurs already during the linear sweep voltammetry that is performed to precondition the electrode before the first double layer experiment is performed.

The ECSA was also tracked for the same samples before and after cyclic voltammetry discussed in the section above (**Figure S13b**). Contrary to CO2RR, the ECSA is unaffected by cyclic voltammetry in the applied potential window (-0.4 V < E_{RHE} < 1.1 V). This corroborates the morphological stability of the core-shell catalysts at -0.6 V v RHE in the main manuscript and the increased stability that all the shells provide the underlying Cu NS. The electrochemical surface area agrees with the morphological stability concluded from electron microscopy. All shells are able to passivate the underlying Cu, but only the Cu-ZrOx interface is stable at CO2RR potentials, resulting in a remarkable morphological stability and stable methane production over multiple hours.

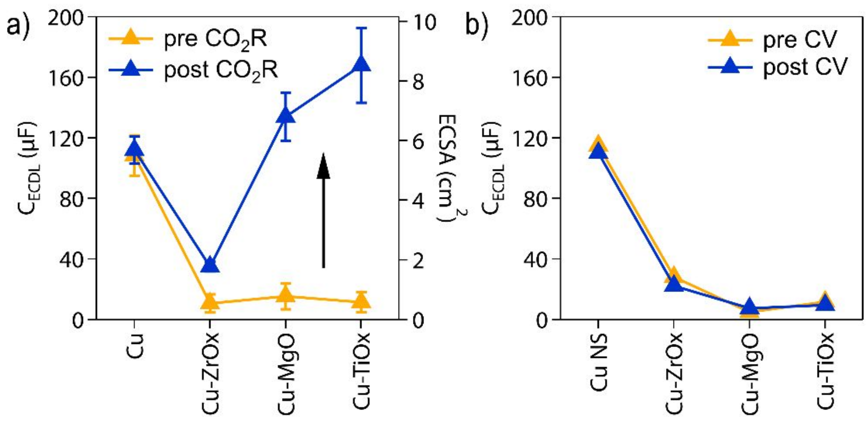

Figure S13: Electrochemical surface area estimated from the charging current in an electrochemical double layer experiment a) before and after CO2RR, and b) before and after cyclic voltammetry between -0.4 and 1.1 V vs RHE. Error bars represent the standard deviation from at least 5 different CO2RR experiments on the respective catalysts.

S2.8 Chronoamperometry at -0.6 V vs RHE

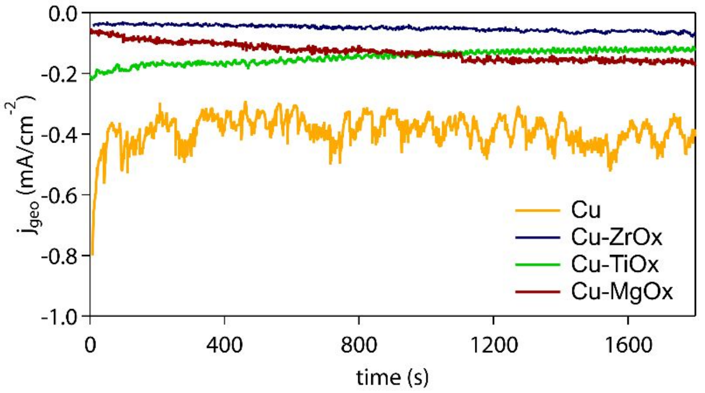

Figure S14: 30 minute chronoamperometry traces for bare Cu nanospheres and the 3 core-shell systems Cu-ZrOx, Cu-TiOx and Cu-MgOx at -0.6 V vs RHE. The traces represent electrochemistry data of the electron microscopy data presented in the main text.

S2.9 Mg ions in the electrolyte

The dissolution of Mg in neutral electrolyte is predicted by the Pourbaix diagram. To test whether dissolved Mg cations could influence the CO2RR selectivity we performed chronoamperometry in electrolyte containing 1 mM Mg ions. No significant change in the product distribution is identified when comparing the Cu+MgOx physical mixtures in 0.1 M $KHCO_3$ with the same Cu nanoparticles in electrolyte spiked with 1 mM of Mg (**Figure S15**). At most, the contribution of the Mg-ions in solution could be reflected in a slight increase of the faradaic efficiency of the hydrogen evolution reaction when compared to the bare Cu presented in Mg-free electrolyte in the main manuscript. The geometric current densities of both samples were comparable at an average -8.0 and -9.4 mA.cm^{-2}.

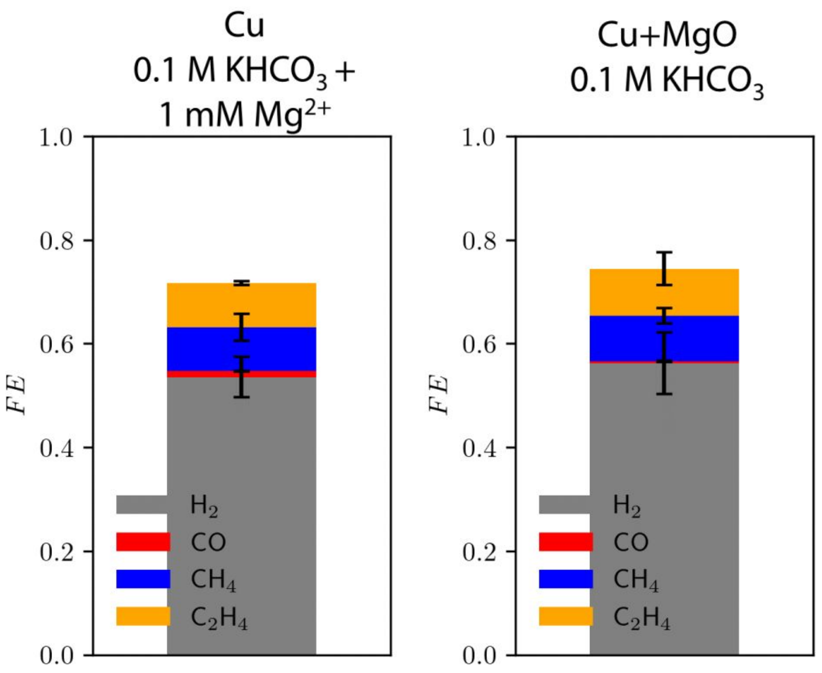

Figure S15: Faradaic efficiency for gas products evolved over a physical mixture of Cu and MgO and the same Cu nanoparticles undergoing electrolysis in the same electrolyte spiked with 1 mM of Mg-ions.

S2.10 Post-electrolysis electron microscopy on Cu-TiOx and Cu+TiOx physical mixtures

To corroborate the identical electrocatalytic behavior of core-shell and physical mixtures between Cu and TiOx, the post mortem morphology was evaluated with electron microscopy. Although highly irregular in both cases, a similar reconstructed catalyst was retrieved for both samples, reinforcing the notion that independent of the starting morphology, Cu and TiOx evolve to form a reconstructed mixed phase catalyst during operation (**Figure S16**). The evolution towards a morphologically similar active catalyst and their identical catalytic behavior hint at a potential dependent electrochemically active surface. In the case of the core-shell particles, some individual smaller particles can also be seen on the microscopy images. Their small size and low contrast make a reliable size estimate difficult, but diameters between 2 and 3 nm suggest that they are much smaller than the initial core-shell particles. The remaining smaller particles could arise from a delayed and slower reconstruction observed also in experiments at less negative potential, cyclic voltammetry and the gradual reduction of Cu observed for Cu-TiOx in XAS.

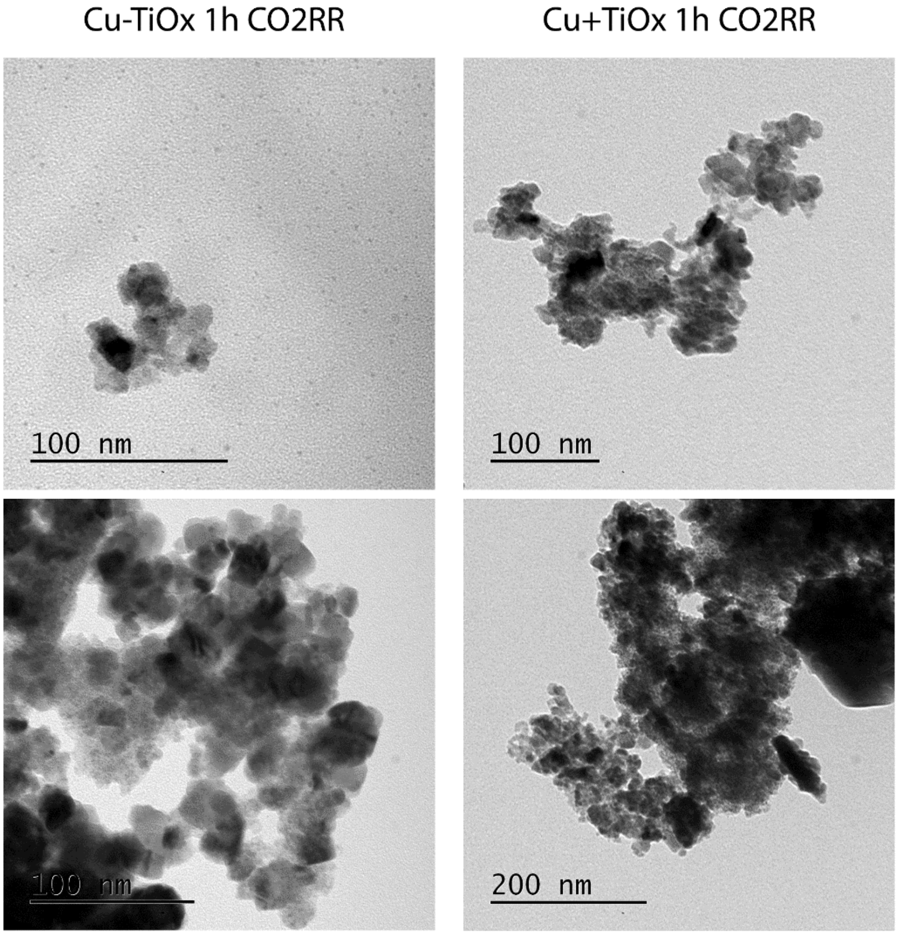

Figure S16: Post-mortem electron microscopy of core-shell and physical mixtures of catalysts containing Cu and TiOx.

S2.11 Current density normalized by electrochemically active surface area

The data presented in Figure 2b of the main manuscript is presented below as a table for referencing:

j_{ECSA} (mA.cm^{-2})	H_2	CO	CH_4	C_2H_4
**Cu NS**	0.783	0.020	0.176	0.339
**Cu-ZrOx**	1.462	0.031	0.368	0.066
**Cu-MgOx**	0.554	0.107	0.044	0.038
**Cu-TiOx**	3.250	0.001	0.001	0.000

Table S1: Product specific current density normalized by ECSA for the 4 catalysts presented in main manuscript Figure 2b.

S3 X-ray absorption spectroscopy

S3.1 X-ray absorption spectra near the Cu K-edge at decreasing potentials

X-ray absorption near edge spectra are provided here for each of the tested potentials reported in the main manuscript (**Figure S17**). Each spectrum displayed here corresponds to the average of all spectra recorded during the 1 hour time interval at the respective potential. This approach was needed to achieve an acceptable signal to noise level for our experiments with low Cu loading on the flat glassy carbon electrode surface.

The trends discussed in the main manuscript can be concluded qualitatively also here from the spectra. Whereas the Cu NS spectrum changes to a predominantly metallic spectrum immediately at -0.2 V, all core-shell systems change more gradually. In Cu-MgOx, the appearance of metallic features is only apparent at -0.8 V. In Cu-ZrOx, the characteristic second peak at 9007 eV remains most heavily suppressed, whereas Cu-TiOx shows a gradual evolution towards a final spectrum that looks predominantly metallic.

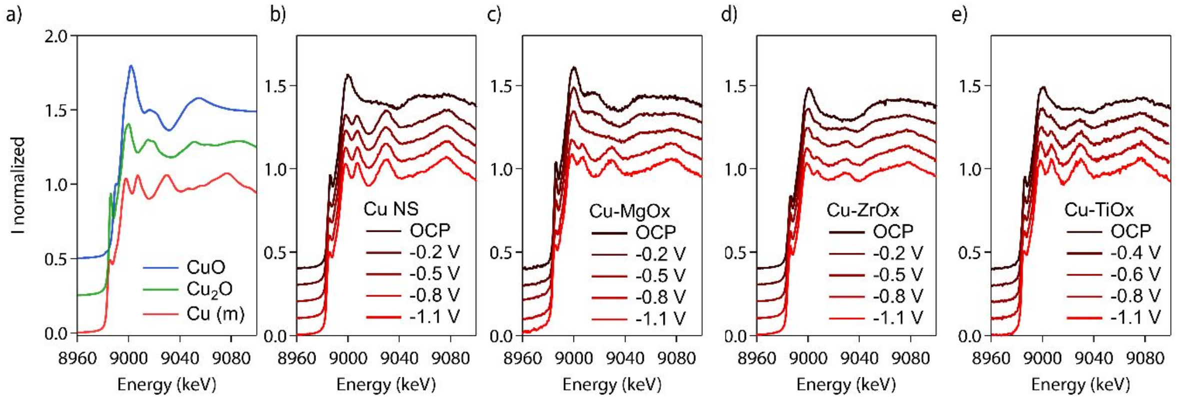

Figure S17: Normalized operando x-ray absorption spectra near the Cu K-edge at different cathodic potential for the a) reference materials, b) Cu NS, c) Cu-MgOx NS, d) Cu-ZrOx and e) Cu-TiOx.

S3.2 XANES analysis

Linear combination analysis with standard reference materials was employed as the primary method to extract quantitative information on the Cu speciation during electrochemistry. Different models are created by linearly combining the reference spectra. With a least-squares fitting the coefficients of each model are adjusted to match the experimental data. The model choice and extracted mole fractions are evaluated based on the match between the fitted models and the experimental data and are further confirmed by performing a principal component analysis followed by back-transformation onto the reference samples.

Representative examples of the fitting models at different potentials is provided for the Cu nanospheres (**Figure S18**). Three models are tested at open circuit potential. Fit #01 describes the data accurately, combining both metallic Cu, Cu(I) and Cu(II) (**Figure S18a**). When either Cu(II) or Cu(m) are excluded from the fit, the fit quality deteriorates significantly (**Figure S18b and c**). At -1.1 V the choice of model is less clear cut, because the sample is predominantly metallic. Including either Cu(I) or Cu(II) provides a slight improvement of the fit, but at mole fractions of 3 and 1% we cannot conclude their statistical significance. This is further confirmed by obtaining a near identical fit quality (χ^2) when performing the fit with only metallic Cu.

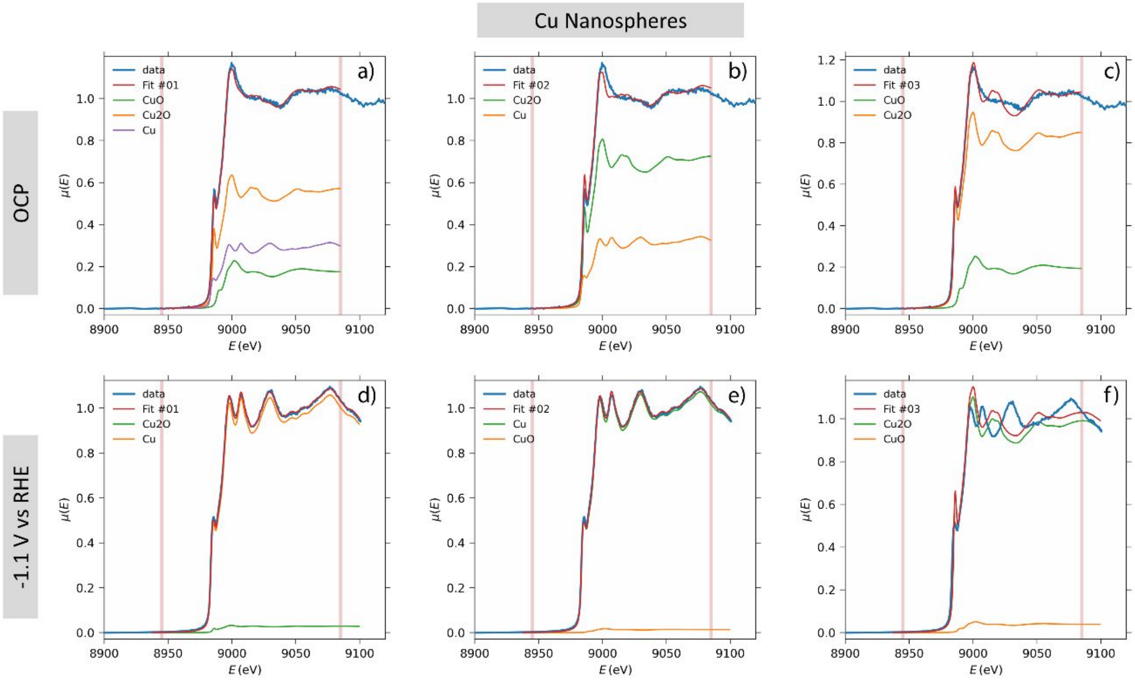

Figure S18: Examples of the linear combination fits with different models at a-c) open circuit potential and d-f) -1.1 V vs RHE. The fits at OCP demonstrate that the best model is quite easily identified by the most accurate reproduction of the experimental spectrum. The spectra at -1.1 V indicate that with 1 dominant contribution (here Cu^0), the LCA can not distinguish whether the second contribution arises from Cu(I) or Cu(II), and an almost identical fit can be achieved by using only the metallic reference spectrum. From this example we conclude that the presence of oxidic Cu is negligible in pure Cu NS at -1.1 V.

As a second example, the best fits of the Cu-ZrOx sample at decreasing bias are presented (**Figure S19**). Here, all fits at cathodic bias yield a good match with the experimental data. We conclude that the reference materials of Cu foil, Cu_2O and CuO provide a good fit of the catalyst near edge structure under operation. Removing any of the three components at small negative bias yielded here a large increase in χ^2, and the contribution of Cu(II) at -1.1 V remains crucial to improve the fit. The importance of all three Cu species (Cu0, Cu(I), Cu(II)) in Cu-ZrOx is further corroborated by principal component analysis. With the principal components of Cu-ZrOx during the potential sweep, the reference spectrum of all three Cu standards CuO can be recreated, demonstrating that the individual components vary independently throughout the electrochemical experiments and are all present at some point during the analysis, validating their use in the LCA (**Figure S20**).

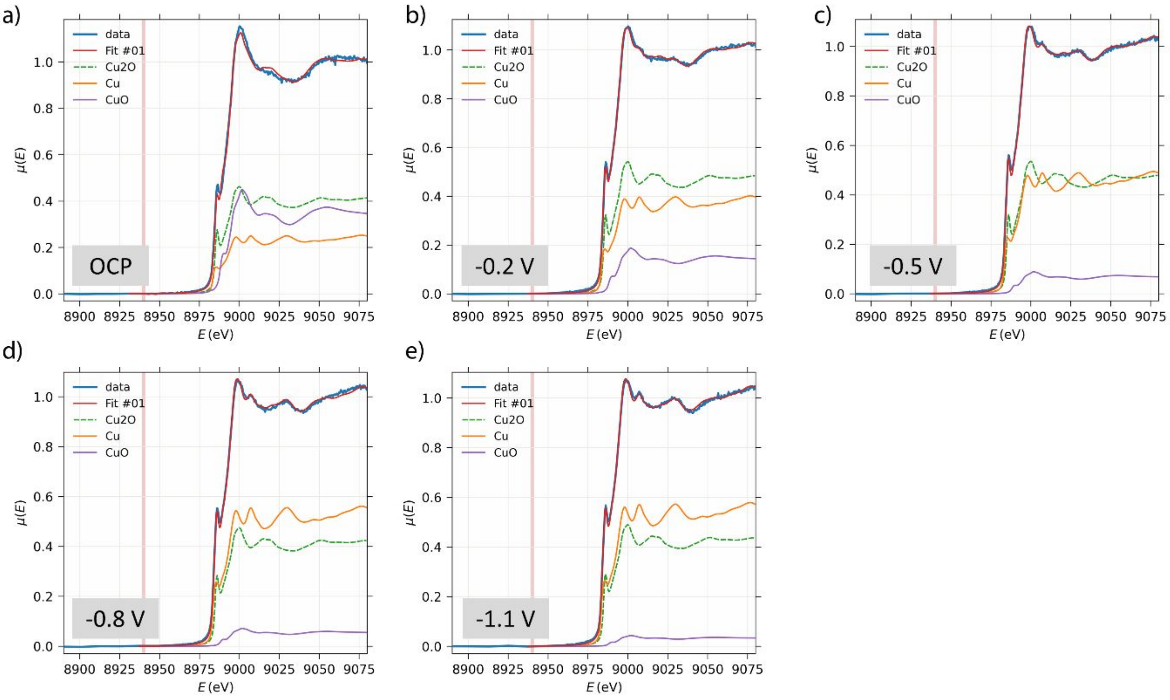

Figure S19: LCA fits of Cu-ZrOx at decreasing potentials. Fit lines in red are given alongside coefficient weighted reference spectra that make up the fit line. Potentials are given versus RHE.

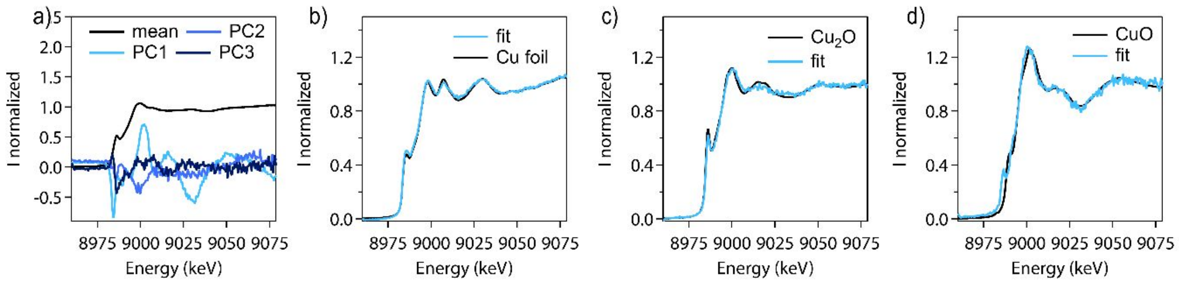

Figure S20: Principal component analysis of the Cu-ZrOx sample during electrochemistry. a) Principal components computed from the XANES spectra of Cu-ZrOx at different potentials. Back-transformation of the principal components onto the reference spectra of b) Cu-foil, c) Cu_2O and d) CuO.

S3.3 Evolution of the Cu K-edge near edge region at -1.1 V vs RHE

Despite the time-averaged Cu K-edge spectrum of Cu-MgOx at -1.1 V showing a persistent oxidic fraction, information with respect to the temporal evolution is lost when averaging over the entire time at each potential step. When instead the intensity of the white line is compared to the intensity of the characteristic metallic feature at 9007 eV for each spectrum, it becomes apparent that Cu in Cu-MgOx keeps evolving towards an entirely metallic structure below -0.8 V (**Figure S21a**). The same can be concluded from the linear combination analysis when the spectra are averaged per 15-minute time interval (**Figure S21b and c**). The Cu speciation in Cu-ZrOx does not change at -1.1 V as a function of time, but Cu in Cu-MgOx keeps being reduced until reaching a near unity Cu^0 mole fraction.

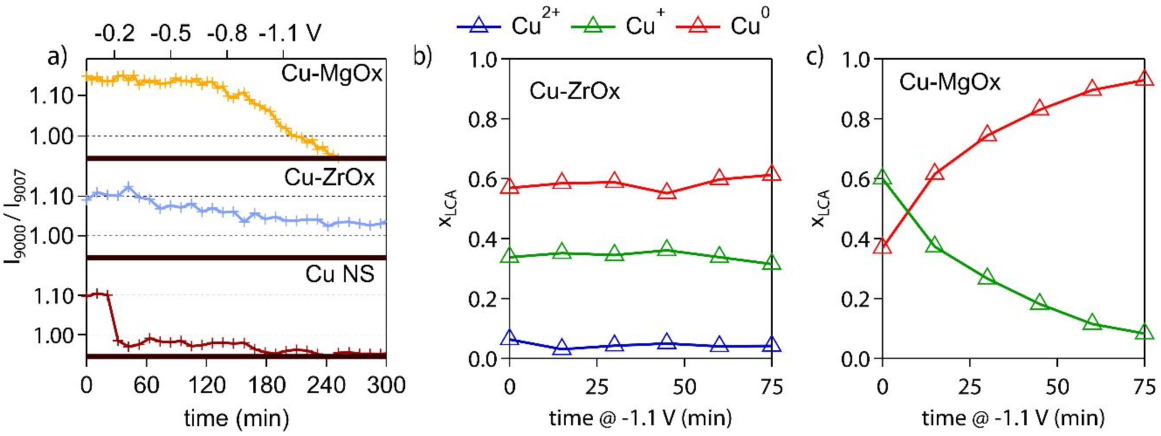

Figure S21: a) Relative intensity between the characteristic white line of Cu and the metallic feature at 9007 eV. A decrease of the ratio occurs when oxides are reduced to form metallic Cu. Speciation extracted from LCA fitting of the spectra at 15 minute time intervals at -1.1 V vs RHE for b) Cu-ZrOx and c) Cu-MgOx. The data indicates the stability of the speciation in Cu-ZrOx contrasting the continuing reduction of Cu in Cu-MgOx at CO2RR potentials until an nearly entirely metallic sample is obtained.

S3.4 Zr K-edge X-ray absorption near edge structure

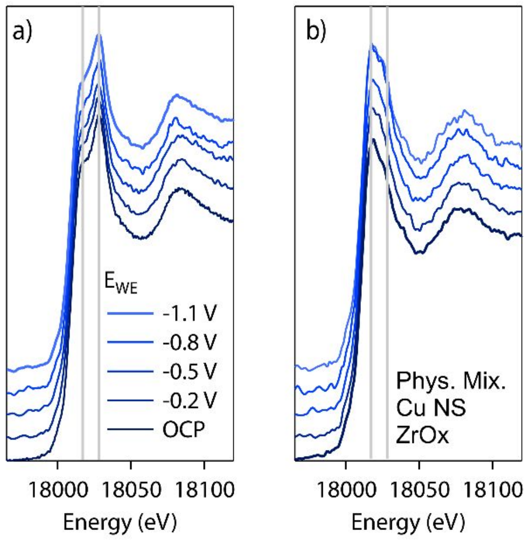

Figure S22: Operando Zr K-edge XANES spectra for a) Cu-ZrOx core-shell nanocrystals and b) Cu + ZrOx physical mixture. Color code for potentials applied to both panels.

The near edge region of the Zr K-edge is rich in information and a qualitative interpretation is possible from the relative intensities of the low-energy and high-energy features in the white line absorption. [7–9] In solids where the Zr-atom displays a higher oxygen coordination of 8 such as in zircon, the low energy feature is more pronounced than the high energy side of the white line. As the coordination number of Zr lowers to 6 as in octahedral coordination, the trend inverts, yielding a step like absorption at the Zr-edge with a lower intensity feature followed by a higher intensity feature at high energy. [7–9] The body of literature on Zr-oxide x-ray absorption studies allows us to confidently conclude that the coordination number of Zr in Cu-ZrOx is better represented by a 6-fold, octahedral coordination (**Figure S22a**). In contrast, the separate preparation of Zr-oxide followed by physical mixing results in a more typical 8-fold coordination (**Figure S22b**). These data are interesting because they evidence the impact of creating an interface on the local coordination geometry of the oxide. Herein, both coordination geometries are stable during CO2RR. However, this detail is important to keep in mind for interpreting the behavior of M|MOx interfaces in future studies.

S3.5 Cu K-edge of Cu+ZrOx physical mixture at -1.1 V vs RHE

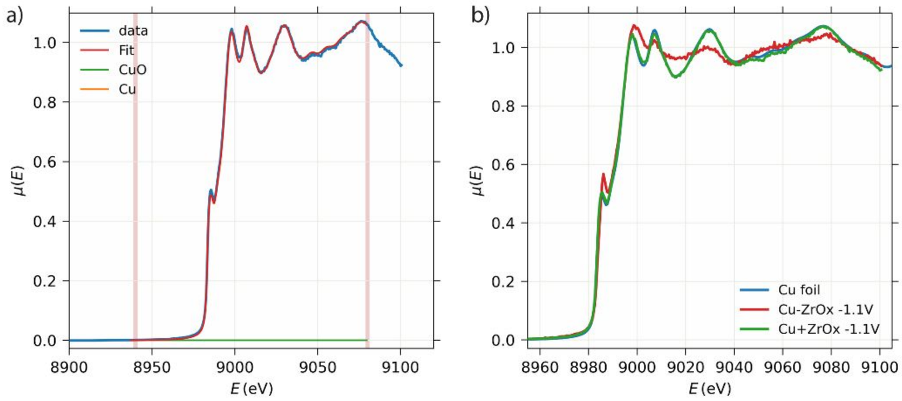

Figure S23: a) LCA for the Cu+ZrOx physical mixture at -1.1 V vs RHE. The fit indicates that only metallic Cu is needed to accurately reproduce the operando spectrum. b) Comparison of a Cu foil XANES spectrum with Cu-ZrOx core-shell and Cu + ZrOx physical mixture at -1.1 V.

Bibliography

1. Albertini, P. P. *et al.* Hybrid oxide coatings generate stable Cu catalysts for CO2 electroreduction. *Nat Mater* **23**, 680–687 (2024).

2. Loiudice, A., Strach, M., Saris, S., Chernyshov, D. & Buonsanti, R. Universal Oxide Shell Growth Enables in Situ Structural Studies of Perovskite Nanocrystals during the Anion Exchange Reaction. *J Am Chem Soc* **141**, 8254–8263 (2020).

3. Loiudice, A., Segura Lecina, O., Bornet, A., Luther, J. M. & Buonsanti, R. Ligand Locking on Quantum Dot Surfaces via a Mild Reactive Surface Treatment. *J Am Chem Soc* **143**, 13418–13427 (2021).

4. Kim, D., Kley, C. S., Li, Y. & Yang, P. Copper nanoparticle ensembles for selective electroreduction of CO2 to C2–C3 products. *Proc Natl Acad Sci U S A* **114**, 10560–10565 (2017).

5. Li, Y. *et al.* Electrochemically scrambled nanocrystals are catalytically active for CO 2-to-multicarbons. *Proceedings of the National Academy of Sciences* **117**, 9194–9201 (2020).

6. Vavra, J., Shen, T. H., Stoian, D., Tileli, V. & Buonsanti, R. Real-time Monitoring Reveals Dissolution/Redeposition Mechanism in Copper Nanocatalysts during the Initial Stages of the CO2 Reduction Reaction. *Angewandte Chemie - International Edition* **60**, 1347–1354 (2021).

7. Filimonova, O. N., Trigub, A. L., Shikina, N. D., Nickolsky, M. S. & Tagirov, B. R. The state of Zr and Hf in chloride hydrothermal fluids from in situ X-ray absorption spectroscopy. *Chem Geol* **641**, (2023).

8. Finkeldei, S. *et al.* Insights into the fabrication and structure of plutonium pyrochlores. *J Mater Chem A Mater* **8**, 2387–2403 (2020).

9. Mckeown, D. A., Muller, I. S., Buechele, A. C. & Pegg, I. L. *X-Ray Absorption Studies of the Local Environment of Zr in High-Zirconia Borosilicate Glasses*. www.elsevier.com/locate/jnoncrysol.
